# Investigating the effects of chronic perinatal alcohol and combined nicotine and alcohol exposure on dopaminergic and non-dopaminergic neurons in the VTA

**DOI:** 10.1038/s41598-021-88221-8

**Published:** 2021-04-22

**Authors:** Tina Kazemi, Shuyan Huang, Naze G. Avci, Yasemin M. Akay, Metin Akay

**Affiliations:** Department of Biomedical Engineering, University of Houston, Houston, TX 77204 USA

**Keywords:** Reward, Neurodegeneration, Neurodegenerative diseases, Neurodevelopmental disorders

## Abstract

The ventral tegmental area (VTA) is the origin of dopaminergic neurons and the dopamine (DA) reward pathway. This pathway has been widely studied in addiction and drug reinforcement studies and is believed to be the central processing component of the reward circuit. In this study, we used a well-established rat model to expose mother dams to alcohol, nicotine-alcohol, and saline perinatally. DA and non-DA neurons collected from the VTA of the rat pups were used to study expression profiles of miRNAs and mRNAs. miRNA pathway interactions, putative miRNA-mRNA target pairs, and downstream modulated biological pathways were analyzed. In the DA neurons, 4607 genes were differentially upregulated and 4682 were differentially downregulated following nicotine-alcohol exposure. However, in the non-DA neurons, only 543 genes were differentially upregulated and 506 were differentially downregulated. Cell proliferation, differentiation, and survival pathways were enriched after the treatments. Specifically, in the PI3K/AKT signaling pathway, there were 41 miRNAs and 136 mRNAs differentially expressed in the DA neurons while only 16 miRNAs and 20 mRNAs were differentially expressed in the non-DA neurons after the nicotine-alcohol exposure. These results depicted that chronic nicotine and alcohol exposures during pregnancy differentially affect both miRNA and gene expression profiles more in DA than the non-DA neurons in the VTA. Understanding how the expression signatures representing specific neuronal subpopulations become enriched in the VTA after addictive substance administration helps us to identify how neuronal functions may be altered in the brain.

## Introduction

Alcohol and nicotine are both toxic and psychoactive substances with dependence-producing tendencies^1–3^. Their consumptions are highly linked to temporary behavioral changes, and when used together, they could result in synergistic adverse effects^4,5^. The rate of nicotine use has a positive correlation to the rate of alcohol use disorder (AUD)^6–8^. Substance use among pregnant women continues to be a major public health concern; 5% of pregnant women reported the use of one or more addictive substances during pregnancy and/or breastfeeding^9^. These substances can easily pass to the offspring through both the placenta and breastmilk. According to the Centers for Disease Control and Prevention (CDC), in the year 2016, 1 in 14 women who gave birth in the United States smoked cigarettes during pregnancy^10^. This number could be higher as many women may not report the use of any drugs during pregnancy. The CDC also reported that drinking and binge drinking by pregnant women in the years 2015–2017 were 11.5% and 3.9%, respectively^11^. Therefore, understanding the molecular mechanisms underlying the risks and alterations that occur in the offspring following perinatal drug exposure is crucial to identify potential therapies.

Consumption of alcohol during pregnancy can cause many adverse immediate as well as long-term effects in the fetus, including fetal alcohol spectrum disorders (FASDs), preterm birth, stillbirth, poor coordination, learning disabilities, and neurobehavioral deficits^12–15^. Full-term pregnancy exposure to alcohol disrupts the regulation of neurotransmitters including dopamine (DA), serotonin, glutamate, noradrenaline, acetylcholine, and histamine^16–19^. Young postnatal rodents exposed to prenatal alcohol showed a familiarized response and enhanced preference to alcohol odor^20–29^. They also exhibited increased alcohol consumption which persisted throughout maturation and into their adolescence^20–29^.

Perinatal exposure to nicotine has been implicated with cognitive dysfunction, learning disabilities, sudden infant death disorder (SIDS), alteration within brain cell development, and neurodevelopmental changes at the cellular level^30–35^. Neurotransmitter functions are desensitized upon gestational nicotine exposure with a decreased nicotinic acetylcholine receptors (nAChRs) subunit expression, decrease in the number of dopaminergic neurons within the ventral tegmental area (VTA) and decreased serotonin turnover^30,36–38^. Additionally, animal models have shown that when adolescent and adult rats were prenatally exposed to nicotine, they will self-administer nicotine at larger amounts compared to the rats that were not prenatally exposed^38–41^.

The mesocorticolimbic pathway, also known as the reward pathway, is a network of DA neurons and has been implicated for its involvement in the rewarding properties of natural stimuli as well as drugs of abuse. Addictive drugs, including alcohol and nicotine, increase mesocorticolimbic activity through activation of dopamine neurons. The mesocorticolimbic pathway connects the VTA to the striatum, nucleus accumbens (NAc), and prefrontal cortex (PFC). In the VTA, nicotine directly binds to and activates nAChRs, which are ligand-gated ion channels found in the central and peripheral nervous system and suggested to be the common biological target of nicotine and alcohol^42–46^. Nicotine also indirectly activates DA neurons and enhances extracellular DA in the NAc^42,47,48^ through glutamate and γ-aminobutyric acid (GABA) neurons. Alcohol indirectly excites DA neurons within the VTA by activating VTA nAChRs and depressing GABAergic neuronal firing in the VTA through the endogenous opioid system^44,49–51^. The VTA has mainly of DA, GABA, and glutamate neurons with some neurons exhibiting combinatorial neurotransmitter characteristics^52^.

MicroRNA (miRNA) is a non-coding, short-segmented RNA that is highly conserved and binds to the 3′ untranslated region (UTR) of its target messenger RNA (mRNA). miRNAs play an important role in regulating gene expression and have been suggested as promising biomarkers for many diseases. A single miRNA potentially targets hundreds of mRNAs, regulating their stability and translation. The role of miRNAs in neurodevelopment, synaptic plasticity, addiction, and many regulatory pathways^53–58^ have been proven as a useful tool to study VTA neurons as well as potential therapy targets.

We recently investigated the influence of maternal nicotine intake on genetic pathways of VTA DA and non-DA neurons in rat pups^33^. Our results showed that the extracellular matrix (ECM) receptor interactions were significantly altered in both DA and non-DA neurons. The PI3K/AKT signaling pathway was significantly enriched in DA neurons with many altered miRNA-gene interactions, but not in the non-DA neurons.

In this study, we have investigated the expression profiles of DA and non-DA VTA neurons following perinatal alcohol and nicotine-alcohol exposure. We identified the alterations in both miRNA and mRNA expression profiles and determined significantly expressed miRNAs and mRNAs for both DA and non-DA neurons in the VTA following each treatment group. Putative miRNA-mRNA validated and predicted target pairs were identified along with the biological pathways altered following perinatal alcohol or nicotine-alcohol exposure in the DA and non-DA neurons in the VTA.

## Results

**Figure 1:**
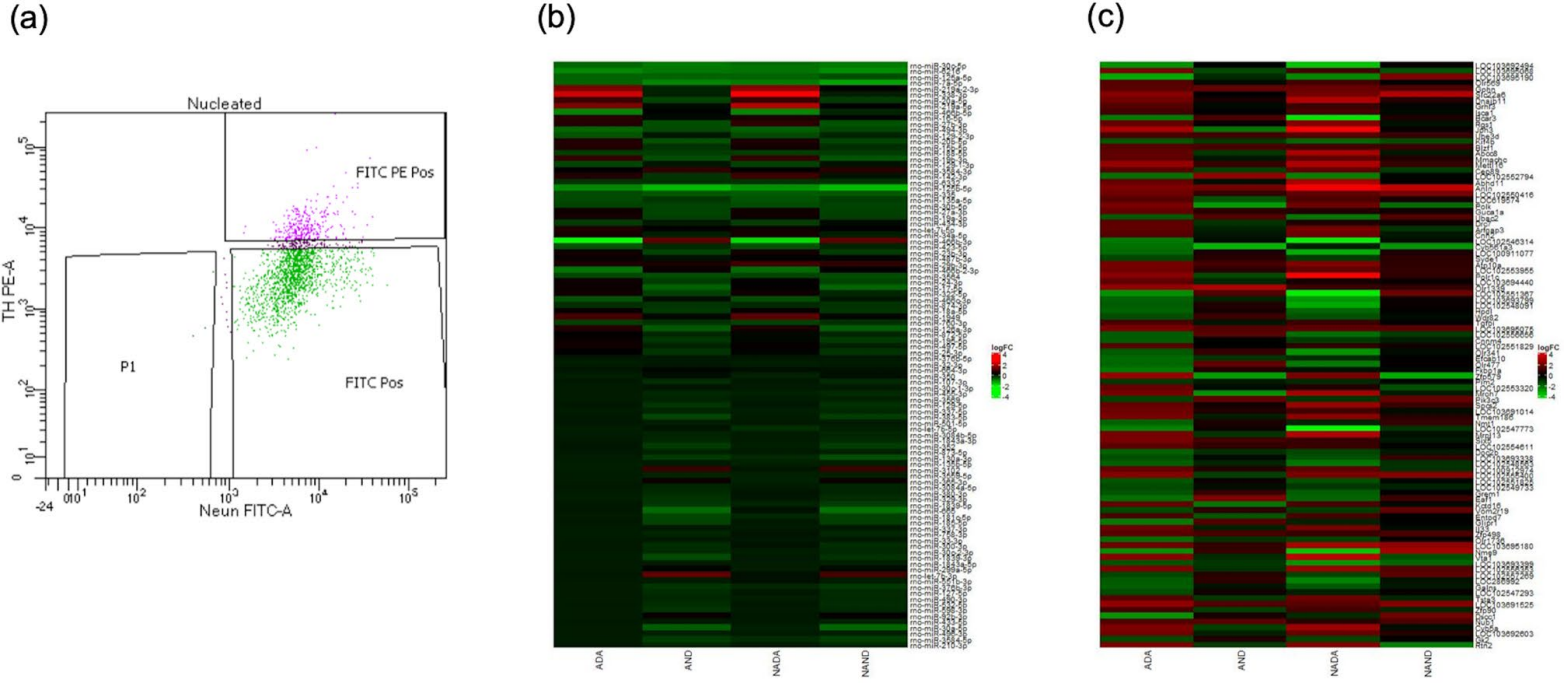
FACS and heat maps following microarray analysis. (**a**) FACS analysis results for DA and non-DA VTA neuron sorting. Heat maps from microarray analysis showing the top 100 (**b**) DEmiRs and (**c**) DEGs for both DA and non-DA VTA neurons following perinatal exposure to alcohol or combined nicotine and alcohol. Expression profiles are based on greatest absolute log fold change.

DA and non-DA VTA neurons were collected from perinatally treated pups exposed to alcohol, combined nicotine-alcohol, and control from gestational day 6 (GD6) to postnatal day 10–14 (PND 10–14). The VTA of offspring was isolated, dissociated, and sorted based on the expression of tyrosine hydroxylase (TH) and neuronal nuclei antibody (NeuN) using fluorescent-activated cell sorting (FACs) (Fig. 1a). Agilent Sureprint miRNA and mRNA microarrays were used to compare gene and miRNA expression profiles following perinatal exposure to alcohol, nicotine-alcohol, and control. Figure 1b,c illustrate the heatmaps of the top 100 significantly differentially expressed miRNAs (DEmiRs) and differentially expressed genes (DEGs) following (alcohol treated DA = ADA, alcohol treated non-DA = AND, combined nicotine and alcohol treated DA = NADA, and combined nicotine and alcohol treated non-DA = NAND) respectively.

### Differential mRNA and miRNA expression analysis following perinatal alcohol and nicotine-alcohol exposure of DA and non-DA neurons

Differential expression was calculated for both DA and non-DA VTA neurons for alcohol and nicotine-alcohol perinatal exposure groups against the control group. The gene expression microarray found 4376 unique genes to be differentially upregulated and 4609 were differentially downregulated following alcohol perinatal exposure in the DA neurons. Following alcohol perinatal exposure in the non-DA neurons, 388 genes were differentially upregulated and 843 were differentially downregulated. The gene expression microarray was used to identify 4607 genes to be differentially upregulated and 4682 were differentially downregulated following nicotine-alcohol perinatal exposure in the DA neurons. Following nicotine-alcohol perinatal exposure in the non-DA neurons, 543 genes were differentially upregulated and 506 were differentially downregulated. Differential expression analysis was done using Benjamini and Hochberg (BH) method and differentially expressed genes were recognized based on q-value < 0.05 and an absolute log2 fold change > 1 as previously described in Keller et al.^32,33^.

The miRNA expression microarray found 64 unique miRNAs to be differentially upregulated and 67 were differentially downregulated following nicotine-alcohol perinatal exposure in the DA neurons. Following nicotine-alcohol perinatal exposure in the non-DA neurons, 46 miRNAs were differentially upregulated and 217 were differentially downregulated. The miRNA expression microarray found 55 unique miRNAs to be differentially upregulated and 58 were differentially downregulated following alcohol perinatal exposure in the DA neurons. Following alcohol perinatal exposure in the non-DA neurons, 65 miRNAs were differentially upregulated and 256 were differentially downregulated. The BH method was used for the statistical analysis applying parameters of q-value < 0.05 and an absolute log2 fold change > 0.5 as previously described in Keller et al.^32,33^. Table 1 shows the top 20 significantly upregulated and downregulated DEmiRs following perinatal (a) nicotine-alcohol on DA, (b) nicotine-alcohol on non-DA, (c) alcohol on DA, and (d) alcohol on non-DA exposures. Table 2 lists the top significant upregulated and downregulated DEGs, their description, and the miRNAs their predicted miRNA targets following perinatal (a) nicotine-alcohol on DA, (b) nicotine-alcohol on non-DA, (c) alcohol on DA, and (d) alcohol on non-DA exposures.

**Table 1:** Leading 20 most significantly DEmiRs.

miRNA accession	miRNA name	Log FC	Adj p val	miRNA accession	miRNA name	Log FC	Adj p val
**Upregulated**				**Downregulated**			
**(a) Perinatal nicotine-alcohol exposure (DA)**							
MIMAT0000581	rno-miR-338-3p	2.675656	5.98E−07	MIMAT0000829	rno-miR-125a-5p	− 0.70493	6.82E−07
MIMAT0005446	rno-miR-219a-2-3p	1.663149	5.98E−07	MIMAT0000575	rno-miR-335	− 0.67566	5.22E−06
MIMAT0000798	rno-miR-27b-3p	0.753301	5.22E−06	MIMAT0000806	rno-miR-30b-5p	− 0.49524	3.33E−05
MIMAT0000801	rno-miR-29b-3p	0.905751	1.51E−05	MIMAT0000830	rno-miR-125b-5p	− 1.13673	6.24E−05
MIMAT0017360	rno-miR-582-3p	0.476339	7.78E−05	MIMAT0005301	rno-miR-188-5p	− 0.36085	7.78E−05
MIMAT0017807	rno-miR-3549	0.416401	8.99E−05	MIMAT0000821	rno-miR-99b-5p	− 0.30386	7.78E−05
MIMAT0000788	rno-miR-19b-3p	0.757479	2.38E−04	MIMAT0000804	rno-miR-30c-5p	− 0.84223	7.78E−05
MIMAT0017892	rno-miR-1249	0.34123	2.43E−04	MIMAT0017120	rno-miR-129-1-3p	− 0.39782	3.43E−04
MIMAT0000889	rno-miR-219a-5p	1.994988	2.48E−04	MIMAT0000781	rno-miR-9a-5p	− 1.41289	4.87E−04
MIMAT0000602	rno-miR-20a-5p	0.839255	3.05E−04	MIMAT0000841	rno-miR-135a-5p	− 0.68061	5.44E−04
MIMAT0017852	rno-miR-1949	1.224315	4.38E−04	MIMAT0025073	rno-miR-6332	− 0.23405	5.44E−04
MIMAT0000900	rno-miR-298-5p	0.342978	5.44E−04	MIMAT0005278	rno-miR-466b-5p	− 1.10064	6.28E−04
MIMAT0035734	rno-miR-193b-3p	0.389912	5.99E−04	MIMAT0000601	rno-miR-129-2-3p	− 0.50035	7.22E−04
MIMAT0000815	rno-miR-34a-5p	0.522789	7.22E−04	MIMAT0025048	rno-miR-3099	− 1.03823	1.07E−03
MIMAT0000789	rno-miR-19a-3p	0.453621	8.34E−04	MIMAT0005337	rno-miR-760-3p	− 0.40491	1.09E−03
MIMAT0017798	rno-miR-3544	0.340946	8.34E−04	MIMAT0024856	rno-miR-6216	− 0.86126	1.09E−03
MIMAT0000787	rno-miR-18a-5p	0.358961	8.34E−04	MIMAT0000822	rno-miR-100-5p	− 0.2928	1.35E−03
MIMAT0000799	rno-miR-27a-3p	0.471539	8.36E−04	MIMAT0000807	rno-miR-30d-5p	− 0.3962	1.79E−03
MIMAT0000848	rno-miR-142-3p	0.949395	8.36E−04	MIMAT0017837	rno-miR-3564	− 0.41495	3.31E−03
MIMAT0000793	rno-miR-23b-3p	0.458696	9.75E−04	MIMAT0017840	rno-miR-3065-3p	− 0.35995	3.37E−03
**(b) Perinatal nicotine-alcohol exposure (non-DA)**							
MIMAT0017807	rno-miR-3549	1.414019	1.29E−10	MIMAT0000821	rno-miR-99b-5p	− 0.50306	1.29E−07
MIMAT0017360	rno-miR-582-3p	1.285743	1.13E−09	MIMAT0000859	rno-miR-181b-5p	− 0.75751	2.27E−07
MIMAT0035734	rno-miR-193b-3p	1.188621	4.62E−09	MIMAT0000822	rno-miR-100-5p	− 0.61344	8.43E−07
MIMAT0000900	rno-miR-298-5p	1.003938	4.62E−09	MIMAT0000830	rno-miR-125b-5p	− 1.48984	1.01E−06
MIMAT0017798	rno-miR-3544	1.04078	5.88E−09	MIMAT0000805	rno-miR-30e-5p	− 0.73458	1.11E−06
MIMAT0017855	rno-miR-1188-3p	1.325352	6.00E−09	MIMAT0025065	rno-miR-6326	− 0.39875	1.59E−06
MIMAT0017891	rno-miR-2985	2.236371	7.82E−09	MIMAT0000575	rno-miR-335	− 0.64889	1.81E−06
MIMAT0017146	rno-miR-191a-3p	0.957343	1.07E−08	MIMAT0000820	rno-miR-99a-5p	− 0.74068	2.73E−06
MIMAT0017892	rno-miR-1249	0.722436	3.64E−08	MIMAT0000829	rno-miR-125a-5p	− 0.5261	3.03E−06
MIMAT0003162	rno-miR-1-5p	1.121173	3.64E−08	MIMAT0000796	rno-miR-26a-5p	− 0.72119	3.67E−06
MIMAT0017857	rno-miR-3573-3p	1.031615	6.80E−08	MIMAT0000797	rno-miR-26b-5p	− 0.89006	3.81E−06
MIMAT0017885	rno-miR-702-3p	2.001084	2.27E−07	MIMAT0000781	rno-miR-9a-5p	− 2.07694	3.81E−06
MIMAT0000892	rno-miR-223-3p	1.501847	3.97E−07	MIMAT0017029	rno-miR-328a-5p	− 0.90389	4.05E−06
MIMAT0005319	rno-miR-484	0.467664	9.28E−07	MIMAT0000804	rno-miR-30c-5p	− 0.95268	6.26E−06
MIMAT0003121	rno-miR-483-3p	0.697811	1.11E−06	MIMAT0017157	rno-miR-211-3p	− 0.48915	6.56E−06
MIMAT0003205	rno-miR-409a-3p	1.389242	1.59E−06	MIMAT0000808	rno-miR-30a-5p	− 0.77565	6.56E−06
MIMAT0000898	rno-miR-296-5p	0.446043	1.71E−06	MIMAT0000840	rno-miR-134-5p	− 0.39738	8.34E−06
MIMAT0017805	rno-miR-3085	1.294816	2.73E−06	MIMAT0000798	rno-miR-27b-3p	− 0.60208	8.34E−06
MIMAT0025067	rno-miR-6328	1.539638	3.03E−06	MIMAT0035748	rno-miR-452-5p	− 1.06473	9.67E−06
MIMAT0000594	rno-miR-345-5p	0.459434	3.43E−06	MIMAT0017872	rno-miR-133c	− 0.31743	1.05E−05
**(c) Perinatal alcohol exposure (DA)**							
MIMAT0005446	rno-miR-219a-2-3p	1.506496	2.18E−06	MIMAT0000829	rno-miR-125a-5p	− 0.711	1.83E−06
MIMAT0000581	rno-miR-338-3p	2.22515	3.18E−06	MIMAT0000575	rno-miR-335	− 0.50699	1.43E−04
MIMAT0000798	rno-miR-27b-3p	0.662927	2.61E−05	MIMAT0005301	rno-miR-188-5p	− 0.35901	1.43E−04
MIMAT0000788	rno-miR-19b-3p	0.77312	3.30E−04	MIMAT0000830	rno-miR-125b-5p	− 1.024	2.37E−04
MIMAT0000789	rno-miR-19a-3p	0.536441	3.47E−04	MIMAT0000806	rno-miR-30b-5p	− 0.3971	3.30E−04
MIMAT0000602	rno-miR-20a-5p	0.842434	3.56E−04	MIMAT0017120	rno-miR-129-1-3p	− 0.42087	3.36E−04
MIMAT0000779	rno-let-7i-5p	0.553422	4.59E−04	MIMAT0000804	rno-miR-30c-5p	− 0.73687	3.47E−04
MIMAT0000787	rno-miR-18a-5p	0.382159	7.39E−04	MIMAT0000601	rno-miR-129-2-3p	− 0.56753	3.56E−04
MIMAT0005282	rno-miR-872-5p	0.278079	8.72E−04	MIMAT0024856	rno-miR-6216	− 1.05863	3.56E−04
MIMAT0000801	rno-miR-29b-3p	0.562388	8.72E−04	MIMAT0025073	rno-miR-6332	− 0.2328	7.39E−04
MIMAT0017360	rno-miR-582-3p	0.357025	8.72E−04	MIMAT0005278	rno-miR-466b-5p	− 1.08252	8.72E−04
MIMAT0017807	rno-miR-3549	0.319616	8.72E−04	MIMAT0005284	rno-miR-874-3p	− 0.20061	9.29E−04
MIMAT0000815	rno-miR-34a-5p	0.513356	9.35E−04	MIMAT0005337	rno-miR-760-3p	− 0.3922	1.72E−03
MIMAT0003211	rno-miR-20b-5p	0.626081	1.10E−03	MIMAT0000821	rno-miR-99b-5p	− 0.20099	1.72E−03
MIMAT0003383	rno-miR-497-5p	0.468985	1.10E−03	MIMAT0017837	rno-miR-3564	− 0.45005	1.80E−03
MIMAT0017892	rno-miR-1249	0.272806	1.28E−03	MIMAT0025048	rno-miR-3099	− 0.96111	1.87E−03
MIMAT0001619	rno-miR-322-5p	0.445791	1.28E−03	MIMAT0003193	rno-miR-494-3p	− 0.72635	2.62E−03
MIMAT0000799	rno-miR-27a-3p	0.446902	1.46E−03	MIMAT0000841	rno-miR-135a-5p	− 0.53688	2.62E−03
MIMAT0000870	rno-miR-195-5p	0.410136	1.59E−03	MIMAT0017840	rno-miR-3065-3p	− 0.35783	3.40E−03
MIMAT0000784	rno-miR-15b-5p	0.612112	1.72E−03	MIMAT0000781	rno-miR-9a-5p	− 1.03675	3.62E−03
**(d) Perinatal alcohol exposure (non-DA)**							
MIMAT0017807	rno-miR-3549	1.471797	7.36E−11	MIMAT0000829	rno-miR-125a-5p	− 0.72243	1.21E−07
MIMAT0017360	rno-miR-582-3p	1.429087	2.61E−10	MIMAT0000821	rno-miR-99b-5p	− 0.49538	1.35E−07
MIMAT0035734	rno-miR-193b-3p	1.346621	8.54E−10	MIMAT0000859	rno-miR-181b-5p	− 0.74354	2.38E−07
MIMAT0000900	rno-miR-298-5p	1.103701	1.26E−09	MIMAT0000822	rno-miR-100-5p	− 0.65169	3.45E−07
MIMAT0017798	rno-miR-3544	1.127038	1.98E−09	MIMAT0000830	rno-miR-125b-5p	− 1.46521	1.07E−06
MIMAT0017146	rno-miR-191a-3p	1.070273	3.10E−09	MIMAT0000575	rno-miR-335	− 0.65714	1.42E−06
MIMAT0017855	rno-miR-1188-3p	1.375406	3.10E−09	MIMAT0025065	rno-miR-6326	− 0.38745	1.77E−06
MIMAT0017891	rno-miR-2985	2.350993	3.46E−09	MIMAT0000805	rno-miR-30e-5p	− 0.66068	2.78E−06
MIMAT0003162	rno-miR-1-5p	1.251792	9.17E−09	MIMAT0000796	rno-miR-26a-5p	− 0.71174	4.18E−06
MIMAT0017892	rno-miR-1249	0.795401	9.44E−09	MIMAT0017872	rno-miR-133c	− 0.34677	4.81E−06
MIMAT0017857	rno-miR-3573-3p	0.987721	1.21E−07	MIMAT0017029	rno-miR-328a-5p	− 0.88587	5.02E−06
MIMAT0000892	rno-miR-223-3p	1.663592	1.21E−07	MIMAT0000804	rno-miR-30c-5p	− 0.96724	5.22E−06
MIMAT0017885	rno-miR-702-3p	2.014488	1.85E−07	MIMAT0017157	rno-miR-211-3p	− 0.49951	5.25E−06
MIMAT0000898	rno-miR-296-5p	0.546114	1.85E−07	MIMAT0017321	rno-miR-652-5p	− 0.58823	6.07E−06
MIMAT0005319	rno-miR-484	0.495535	3.96E−07	MIMAT0000817	rno-miR-93-5p	− 0.65507	7.33E−06
MIMAT0017805	rno-miR-3085	1.45774	7.81E−07	MIMAT0000840	rno-miR-134-5p	− 0.3959	8.47E−06
MIMAT0035726	rno-miR-149-5p	0.826697	1.09E−06	MIMAT0000797	rno-miR-26b-5p	− 0.79887	1.02E−05
MIMAT0003121	rno-miR-483-3p	0.691476	1.09E−06	MIMAT0005299	rno-miR-181d-5p	− 0.43718	1.15E−05
MIMAT0003205	rno-miR-409a-3p	1.417714	1.13E−06	MIMAT0035748	rno-miR-452-5p	− 1.01943	1.50E−05
MIMAT0025067	rno-miR-6328	1.6699	1.23E−06	MIMAT0017201	rno-miR-483-5p	− 0.6058	1.67E−05

Following perinatal (a) nicotine-alcohol on DA neurons, (b) nicotine-alcohol on non-DA neurons, (c) alcohol on DA neurons, and (d) alcohol on non-DA neurons exposure. Benjamini–Hochberg method was used for the statistical analysis (q value < 0.05, absolute log2 fold change > 0.5).

**Table 2:** Topmost DEGs and their predicted miRNA targets following microarray expression analysis.

Gene symbol	Entrez ID	Log FC	Adj p val	Description	miRNA target
**(a) Perinatal nicotine-alcohol exposure (DA)**					
**Upregulated**					
LOC103690032	103690032	10.4369813	7.74E−12	Insulinoma-Associated Protein 1-Like	
Lypla2	83510	9.942673	1.60E−11	Lysophospholipase 2	rno-miR-125b-5p rno-miR-125a-5p
Oas1b	246268	7.83135311	2.51E−11	2-5 Oligoadenylate Synthetase 1B	
LOC103694925	103694925	7.55081605	3.23E−11	Uncharacterized LOC103694925	
Gnai2	81664	8.46772863	3.96E−11	G Protein Subunit Alpha I2	rno-miR-30E−5p rno-miR-30c-5p rno-miR-30d-5p rno-miR-30b-5p rno-miR-129-2-3p rno-miR-129-1-3p rno-miR-222-3p
Psmc5	81827	8.38986757	6.90E−11	Proteasome 26S Subunit, ATPase 5	
Tprg1l	687090	10.2807927	6.90E−11	Tumor Protein P63 Regulated 1 Like	
Myl6l	362816	8.82578162	2.28E−10	Myosin Light Polypeptide 6	rno-miR-760-3p
LOC102550923	102550923	8.58711892	2.38E−10	Uncharacterized LOC102550923	
Atp2a2	29693	8.72500481	2.63E−10	ATPase Sarcoplasmic/Endoplasmic Reticulum Ca2 + Transporting 2	rno-miR-135a-5p rno-miR-30c-5p rno-miR-30b-5p rno-miR-30e-5p rno-miR-30d-5p
Yipf1	298312	8.55261635	2.68E−10	Yip1 Domain Family Member 1	
LOC102555035	102555035	8.60241579	2.79E−10	Uncharacterized LOC102555035	
Pabpn1	116697	7.38450229	3.05E−10	Poly(A) Binding Protein Nuclear 1	
Atp5g1	29754	7.22544047	3.09E−10	ATP Synthase H + Transporting Mitochondrial Fo Complex Subunit C1 (Subunit 9)	rno-miR-3065-3p
LOC103693859	103693859	9.52946276	3.50E−10	Uncharacterized LOC103693859	
Bnip3l	140923	8.20049158	6.01E−10	BCL2 Interacting Protein 3 Like	rno-miR-384-5p rno-miR-23b-3p rno-miR-27a-3p rno-miR-27b-3p rno-miR-20a-5p rno-miR-106b-5p rno-miR-138-5p rno-miR-30e-5p rno-miR-137-3p rno-miR-30b-5p rno-miR-30c-5p rno-miR-30d-5p rno-miR-26a-5p rno-miR-129-2-3p rno-miR-129-1-3p
Abca2	79248	7.9834934	7.16E−10	ATP Binding Cassette Subfamily A Member 2	rno-miR-9a-5p rno-miR-181d-5p
Ptov1	292888	6.43951123	7.77E−10	PTOV1 Extended AT-Hook Containing Adaptor Protein	rno-miR-494-3p rno-let-7c-5p rno-miR-328a-3p rno-let-7a-5p
**Downregulated**					
Ccdc175	500668	-10.990851	5.66E−09	Coiled-Coil Domain Containing 175	rno-miR-410-3p rno-miR-340-5p
Bdnf	24225	− 9.2937299	9.65E−09	Brain Derived Neurotrophic Factor	rno-miR-384-5p rno-let-7a-1-3p rno-miR-410-3p rno-miR-15b-5p rno-miR-16-5p rno-miR-195-5p rno-miR-497-5p rno-miR-322-5p rno-miR-495 rno-miR-34a-5p rno-miR-219a-5p rno-miR-338-3p
Cysltr1	114099	− 9.8846902	1.08E−08	Cysteinyl Leukotriene Receptor 1	
Defa5	286995	− 10.674451	2.32E−08	Defensin Alpha 5	
LOC102546831	102546831	− 9.5681235	2.32E−08	Uncharacterized LOC102546831	
Haus4	305882	− 9.2976177	2.68E−08	HAUS Augmin Like Complex Subunit 4	rno-miR-384-5p rno-miR-340-5p
LOC100912124	100912124	− 9.4107294	2.82E−08	Uncharacterized LOC100912124	
Stab2	282580	− 7.7544826	3.68E−08	Stabilin 2	
Gstt4	686922	− 8.6807282	4.65E−08	Glutathione S-Transferase Theta 4	
Coprs	290925	− 8.1574747	4.90E−08	Coordinator Of PRMT5 And Differentiation Stimulator	rno-miR-340-5p
LOC100362043	100362043	− 9.2086997	4.92E−08	rCG64252-Like	
**(b) Perinatal nicotine-alcohol exposure (non-DA)**					
**Upregulated**					
Rn18s	100861533	5.25765391	7.73E−08	18S Ribosomal RNA	
LOC102554599	102554599	6.30966307	2.05E−06	Uncharacterized LOC102554599	
Hivep3	313557	5.2702809	2.05E−06	HIVEP Zinc Finger 3	rno-miR-9a-5p
Ank1	306570	4.91512652	2.05E−06	Ankyrin 1	rno-miR-9a-5p rno-miR-153-3p rno-miR-325-3p rno-miR-218a-5p rno-miR-27a-3p rno-miR-27b-3p rno-miR-103-3p rno-miR-181a-5p rno-miR-181d-5p rno-miR-181c-5p rno-miR-181b-5p rno-miR-138-5p rno-miR-125a-5p rno-miR-125b-5p rno-miR-7b
Gramd1c	360717	4.37037279	2.05E−06	GRAM Domain Containing 1C	
LOC102555591	102555591	5.00148603	2.11E−06	Uncharacterized LOC102555591	
Tcf15	296272	4.80941709	2.14E−06	Transcription Factor 15	rno-miR-9a-5p
LOC100912615	100912615	5.19002225	2.14E−06	Transmembrane Protein 19 Like	rno-miR-30c-2-3p rno-miR-134-5p
Acbd4	303577	4.43885281	2.14E−06	Acyl-CoA Binding Domain Containing 4	rno-miR-24-3p
Crispld1	316482	4.28650888	2.45E−06	Cysteine Rich Secretory Protein LCCL Domain Containing 1	rno-miR-381-3p rno-miR-301a-3p rno-miR-130b-3p rno-miR-130a-3p rno-miR-330-3p rno-miR-340-5p rno-miR-101a-3p rno-miR-101b-3p
LOC102549311	102549311	4.91763847	2.86E−06	Uncharacterized LOC102549311	
LOC103694145	103694145	4.32856169	2.86E−06	Uncharacterized LOC103694145	
LOC103692405	103692405	4.52752952	3.33E−06	Uncharacterized LOC103692405	
Cldn15	304388	5.11253929	4.45E−06	Claudin 15	
Hcrtr2	25605	5.03137012	5.23E−06	Hypocretin Receptor 2	rno-miR-30b-5p rno-miR-384-5p rno-miR-30e-5p rno-miR-30a-5p rno-miR-30c-5p rno-miR-340-5p rno-miR-23b-3p rno-miR-23a-3p rno-miR-301a-3p
Pax3	114502	4.84787676	5.55E−06	Paired Box 3	rno-miR-204-3p rno-miR-218a-5p
Il15ra	690369	5.47741684	5.55E−06	Interleukin 15 Receptor Subunit Alpha	rno-miR-7b rno-miR-7a-5p rno-miR-500-3p
Spag7	303260	4.31200186	5.78E−06	Sperm Associated Antigen 7	rno-miR-103-3p rno-miR-30c-5p rno-miR-195-5p rno-miR-15a-5p rno-miR-342-3p
RT1-CE1	309603	4.51323663	5.80E−06	RT1 class I locus CE12	
LOC102553440	102553440	4.8583428	7.34E−06	SET-binding protein-like	
**Downregulated**					
Cux2	288665	− 5.7425292	7.53E−06	Cut Like Homeobox 2	
RGD1563383	317533	− 6.7392315	8.86E−06	Similar to Hypothetical Protein 4930595M18	
Thap1	306547	− 5.3327844	1.79E−05	THAP Domain Containing 1	rno-miR-138-5p rno-miR-23a-5p
LOC690309	690309	− 7.0396693	2.00E−05	Similar to DNA Methyltransferase 3B	
LOC100911498	100911498	− 7.1074276	4.31E−05	Uncharacterized LOC100911498	
Tpm2	500450	− 4.4267726	7.03E−05	Tropomyosin 2	
LOC102551788	102551788	− 5.9909895	1.54E−04	Uncharacterized LOC102551788	
Kpna5	294392	− 4.6198322	1.80E−04	Karyopherin Subunit Alpha 5	
Pck1	362282	− 4.3844261	1.82E−04	Phosphoenolpyruvate Carboxykinase 1	
**(c) Perinatal alcohol exposure (DA)**					
**Upregulated**					
LOC103690032	103690032	10.2424935	9.88E−12	Insulinoma-associated protein 1-like	
Lypla2	83510	9.50049725	2.95E−11	Lysophospholipase 2	rno-miR-125b-5p rno-miR-125a-5p
Oas1b	246268	7.72616633	2.95E−11	2-5 Oligoadenylate Synthetase 1B	
Gnai2	81664	8.45414809	4.08E−11	G Protein Subunit Alpha I2	rno-miR-30c-5p rno-miR-30d-5p rno-miR-30b-5p rno-miR-129-1-3p rno-miR-129-2-3p
Tprg1l	687090	10.3963313	7.19E−11	Tumor Protein P63 Regulated 1 Like	
Psmc5	81827	8.01145292	1.69E−10	Proteasome 26S Subunit, ATPase 5	
LOC103694925	103694925	6.70099446	1.69E−10	Uncharacterized LOC103694925	
Rpl4	64302	7.28851652	1.73E−10	Ribosomal Protein L4	
LOC102555035	102555035	8.82453566	2.19E−10	Uncharacterized LOC102555035	
Myl6l	362816	8.50351604	3.86E−10	Myosin Light Polypeptide 6	rno-miR-760-3p
LOC102553223	102553223	7.93413042	4.11E−10	Uncharacterized LOC102553223	
LOC102553290	102553290	9.41489199	4.22E−10	Collagen Alpha-1(III) Chain-Like	
LOC102550923	102550923	7.93083256	7.18E−10	Uncharacterized LOC102550923	
Pabpn1	116697	6.96315071	7.81E−10	Poly(A) Binding Protein Nuclear 1	
LOC103693859	103693859	8.983498	9.35E−10	Uncharacterized LOC103693859	
Yipf1	298312	7.82293398	1.01E−09	Yip1 Domain Family Member 1	
Bnip3l	140923	7.89536462	1.04E−09	BCL2 Interacting Protein 3 Like	rno-miR-384-5p rno-miR-23b-3p rno-miR-27b-3p rno-miR-27a-3p rno-miR-106b-5p rno-miR-20a-5p rno-miR-30d-5p rno-miR-30b-5p rno-miR-30c-5p rno-miR-26a-5p rno-miR-129-1-3p rno-miR-129-2-3p
RT1-A1	24973	7.82566211	1.04E−09	RT1 class Ia, locus A1	rno-miR-22-5p rno-miR-125b-5p
**Downregulated**					
Defa5	286995	− 11.767855	7.07E−09	Defensin Alpha 5	
Cysltr1	114099	− 10.021291	9.35E−09	Cysteinyl Leukotriene Receptor 1	
Ccdc175	500668	− 10.321499	1.26E−08	Coiled-Coil Domain Containing 175	rno-miR-410-3p rno-miR-340-5p
Bdnf	24225	− 9.0000576	1.48E−08	Brain Derived Neurotrophic Factor	rno-miR-384-5p rno-let-7a-1-3p rno-miR-410-3p rno-miR-15b-5p rno-miR-16-5p rno-miR-195-5p rno-miR-322-5p rno-miR-497-5p rno-miR-495 rno-miR-34a-5p rno-miR-219a-5p rno-miR-338-3p
LOC100362043	100362043	− 9.9434402	2.02E−08	rCG6452-Like	
Zfp68	304337	− 8.7004104	2.33E−08	Zinc Finger Protein 68	rno-miR-340-5p
LOC102546831	102546831	− 9.5438671	2.67E−08	Uncharacterized LOC102546831	
Gstt4	686922	− 9.0942988	2.85E−08	Glutathione S-Transferase Theta 4	
Stab2	282580	− 7.8049797	3.77E−08	Stabilin 2	
Nufip1	364430	− 8.000401	4.53E−08	Nuclear FMR1 Interacting Protein 1	rno-miR-340-5p rno-miR-1-5p rno-miR-27a-3p rno-miR-27b-3p rno-miR-338-3p
Cyp4 × 1	246767	− 8.6281046	5.55E−08	Cytochrome P450 Family 4 Subfamily X Member 1	
**(d) Perinatal alcohol exposure (non-DA)**					
**Upregulated**					
Rn18s	100861533	4.15668112	9.43E−06	18S Ribosomal RNA	
Gramd1c	360717	4.33015223	9.43E−06	GRAM Domain Containing 1C	
LOC102554599	102554599	6.07875332	9.43E−06	Uncharacterized LOC102554599	
Acbd4	303577	4.41524532	9.43E−06	Acyl-CoA Binding Domain Containing 4	rno-miR-24-3p
Tcf15	296272	4.72500301	9.43E−06	Transcription Factor 15	rno-miR-9a-5p
Crispld1	316482	4.26646881	9.43E−06	Cysteine Rich Secretory Protein LCCL Domain Containing 1	rno-miR-381-3p rno-miR-130a-3p rno-miR-301a-3p rno-miR-130b-3p rno-miR-330-3p rno-miR-374-5p rno-miR-340-5p rno-miR-101b-3p rno-miR-101a-3p
LOC103694145	103694145	4.34974398	9.43E−06	Uncharacterized LOC103694145	
Ank1	306570	4.61031314	9.43E−06	Ankyrin 1	rno-miR-103-3p rno-miR-153-3p rno-miR-325-3p rno-miR-27a-3p rno-miR-27b-3p rno-miR-181b-5p rno-miR-181a-5p rno-miR-181d-5p rno-miR-181c-5p rno-miR-138-5p rno-miR-9a-5p rno-miR-125b-5p rno-miR-125a-5p rno-miR-7b
LOC102555591	102555591	4.71876013	1.01E−05	Uncharacterized LOC102555591	
LOC100912615	100912615	4.9216121	1.05E−05	Transmembrane Protein 19-Like	rno-miR-30c-2-3p rno-miR-134-5p
LOC102549311	102549311	4.80451405	1.05E−05	Uncharacterized LOC102549311	
LOC103692405	103692405	4.20749764	2.71E−05	Uncharacterized LOC103692405	
RT1-CE1	309603	4.34803972	3.15E−05	RT1 class I locus CE12	
Hivep3	313557	4.40420951	3.42E−05	HIVEP Zinc Finger 3	rno-miR-9a-5p
Hcrtr2	25605	4.68617264	3.54E−05	Hypocretin Receptor 2	rno-miR-23a-3p rno-miR-340-5p rno-miR-30d-5p rno-miR-30c-5p rno-miR-384-5p rno-miR-30b-5p rno-miR-30a-5p rno-miR-30e-5p rno-miR-23b-3p rno-miR-30c-2-3p rno-miR-301a-3p
Spag7	303260	4.0365198	3.75E−05	Sperm Associated Antigen 7	rno-miR-195-5p rno-miR-103-3p rno-miR-322-5p rno-miR-30c-5p rno-miR-15a-5p rno-miR-342-3p
LOC102556393	102556393	4.72059462	3.75E−05	Uncharacterized LOC102556393	
LOC103694867	103694867	5.61215284	3.79E−05	ADP-ribosylation factor-binding protein GGA2-like	
Pax3	114502	4.43646295	4.37E−05	Paired Box 3	rno-miR-204-3p
LOC103692432	103692432	3.6173433	4.91E−05	Uncharacterized LOC103692432	
**Downregulated**					
Icmt	170818	− 7.1274883	3.54E−05	Isoprenylcysteine Carboxyl Methyltransferase	rno-miR-483-3p
Cux2	288665	− 5.3355855	5.32E−05	Cut Like Homeobox 2	
LOC690309	690309	− 7.1412271	5.59E−05	Similar to DNA Methyltransferase 3B	
RGD1563383	317533	− 6.0939622	7.16E−05	Similar to Hypothetical Protein 4930595M18	
LOC103691640	103691640	− 5.0208823	1.52E−04	Uncharacterized LOC103691640	
Kpna5	294392	− 4.8229431	2.20E−04	Karyopherin Alpha 5	
Kcng1	296395	− 3.6216752	2.87E−04	Potassium Voltage-Gated Channel Modifier Subfamily G Member 1	
Clspn	298534	− 6.7745557	3.31E−04	Claspin	rno-miR-299a-5p
Vom2r-ps20	690123	− 4.5902179	3.41E−04	Vomeronasal 2 Receptor, Pseudogene 20	
Tpm2	500450	− 4.1331145	3.41E−04	Tropomyosin 2	

Most significant upregulated and downregulated DEGs following perinatal (a) nicotine-alcohol exposure on DA neurons, (b) nicotine-alcohol exposure on non-DA neurons, (c) alcohol exposure on DA neurons, and (d) alcohol exposure on non-DA neurons. The genes were listed after Benjamini–Hochberg corrections with adjusted p-value < 0.001, q-value < 0.05, and absolute log2 fold change > 1.

### Integrated analysis of significantly DEmiRs and their significantly DEG targets profiling

**Figure 2:**
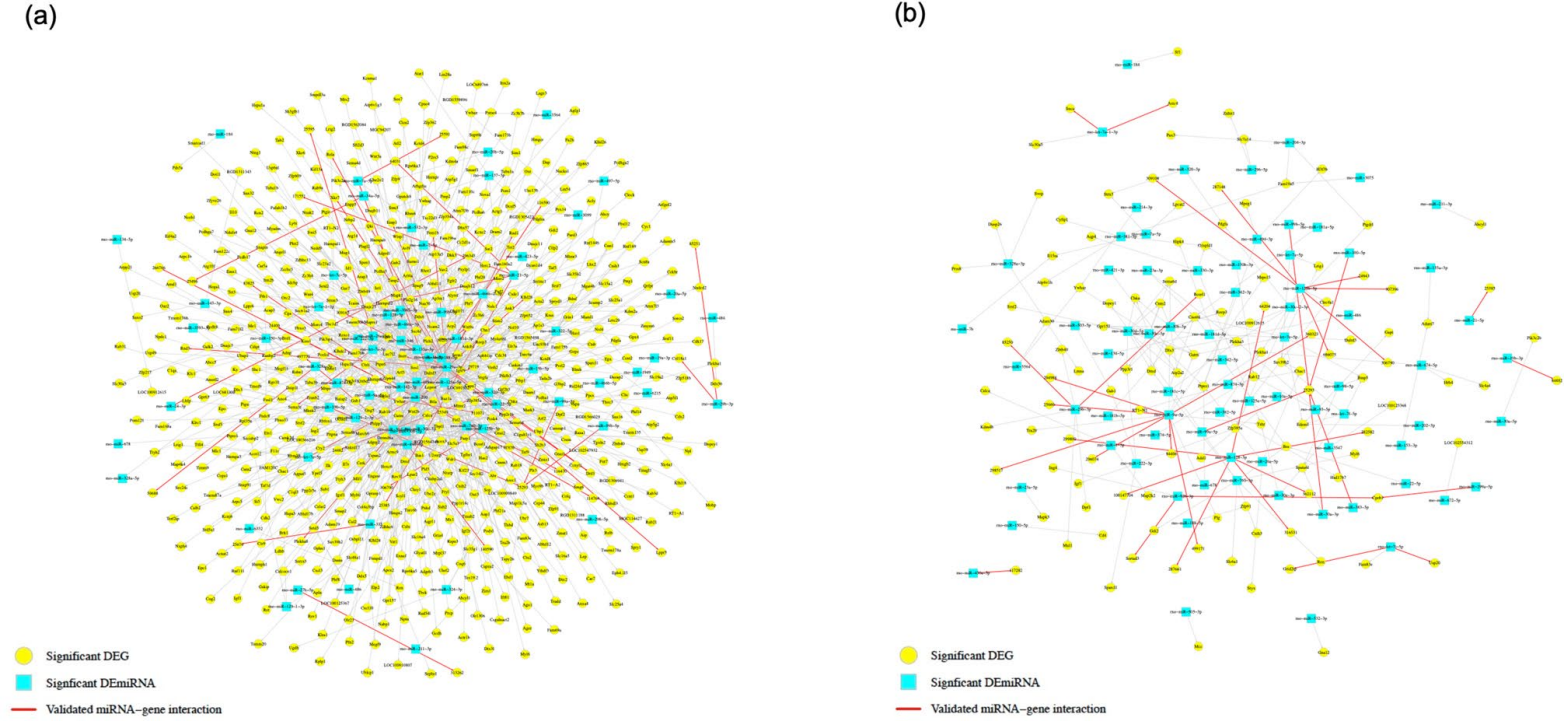
Predicted and validated miRNA-mRNA target network. Using multiMiR, miRNA-mRNA target networks were identified using negative expression correlation following perinatal (**a**) nicotine-alcohol on DA neurons, and (**b**) nicotine-alcohol on non-DA neurons. The node shapes (blue square) denote DEmiRs while (yellow circle) denotes DEGs. Red edges suggest validated miRNA-gene network pairs based on existing evidence from the literature.

**Figure 3:**
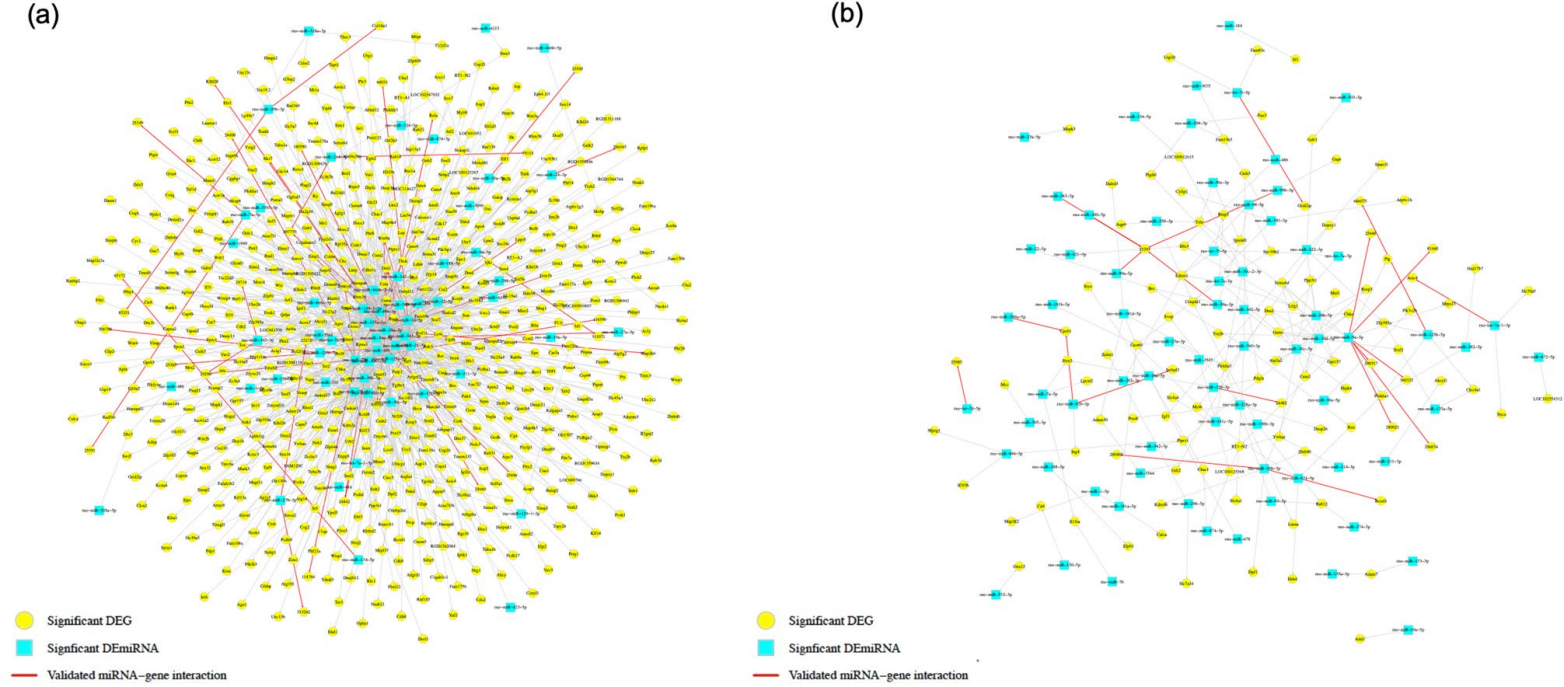
Predicted and validated miRNA-mRNA target network. Using multiMiR, miRNA-mRNA target networks were identified using negative expression correlation following perinatal (**a**) alcohol on DA neurons, and (**b**) alcohol on non-DA neurons. The node shapes (blue square) denote DEmiRs while (yellow circle) denotes DEGs. Red edges suggest validated miRNA-gene network pairs based on existing evidence from the literature.

Significantly DEmiRs and DEGs lists were processed using the MultiMiR^59^ R package to find the predicted and validated mRNA and miRNA targets following each perinatal exposure group. Following perinatal alcohol exposure on DA neurons, a total of 107 unique miRNAs were target paired with 3159 unique genes with 28 validated miRNA-gene pairs. Following perinatal alcohol exposure on non-DA neurons, a total of 158 unique miRNAs were target paired with 421 unique genes with 18 validated miRNA-gene pairs. Following perinatal nicotine-alcohol exposure on DA neurons, a total of 125 unique miRNAs were target paired with 3663 unique genes with 89 validated miRNA-gene pairs. Following perinatal nicotine-alcohol exposure on non-DA neurons, a total of 163 unique miRNAs were target paired with 416 unique genes with 32 validated miRNA-gene pairs. Among the DEmiRs, rno-miR-29b-3p was significantly enriched in all groups, targeting a high number of genes. Figure 2 illustrates the predicted miRNA-gene target networks following perinatal (a) nicotine-alcohol on DA, and (b) nicotine-alcohol on non-DA. Figure 3 shows the predicted miRNA-gene target networks following perinatal (a) alcohol on DA, and (b) alcohol on non-DA exposures.

### Functional enrichment analysis and enriched pathways of the DEGs

DAVID v6.8^60,61^ (Supplementary Table S1) and the ClueGO v2.5.6^62^ plugin for Cytoscape v3.8^63^ (Supplementary Figs. S1, S2, S3, S4, S5, S6, S7, and S8) were used to perform functional enrichment analysis on the significantly DEGs. Gene ontology (GO) biological processes and Kyoto Encyclopedia of Genes and Genomes (KEGG)^64–66^ were both included in the ClueGO analysis using Cytoscape. Among the most enriched biological processes following nicotine-alcohol perinatal exposure on DA neurons are Parkinson disease, Huntington disease, as well as Alzheimer disease (p < 0.0001) for the upregulated DEGs. Parkinson disease, thermogenesis and cytokine-cytokine receptor interaction (p < 0.05) were among the enriched biological processes for the downregulated DEGs following perinatal nicotine-alcohol exposure on DA neurons. Among the most enriched biological processes following perinatal nicotine-alcohol exposure on non-DA neurons are proteoglycans in cancer and axon guidance (p < 0.01) for the upregulated DEGs. For the downregulated DEGs following perinatal nicotine-alcohol exposure on non-DA neurons, olfactory transduction and dilated cardiomyopathy (DCM) were among the enriched processes (p < 0.05). Following perinatal alcohol exposure on DA neurons, Parkinson disease, Huntington disease, as well as Alzheimer disease (p < 0.0001) were among the most enriched biological processes for the upregulated DEGs. For the downregulated DEGs following perinatal alcohol exposure on DA neurons, basal cell carcinoma and signal transduction were the most enriched processes (p < 0.05). Following perinatal alcohol exposure on non-DA neurons, cGMP-PKG signaling pathway, choline metabolism in cancer, and osteoclast differentiation (p < 0.05) were among the most enriched biological processes for the upregulated DEGs. Following perinatal alcohol exposure on non-DA neurons, olfactory transduction, glutamatergic synapse, and circadian entrainment were the most enriched biological processes (p < 0.05).

## Discussion

In this study, we have investigated the miRNome and transcriptome profiles of the rat pups which were perinatally exposed to alcohol or nicotine and alcohol combined and then compared by applying functional enrichment analysis among the DA and non-DA neurons of the VTA following exposures. Our well-established animal model^32,33,67–69^ for perinatal nicotine exposure was used to expose dams to nicotine perinatally for four weeks, equivalent to the full term human pregnancy^19,70–73^. A Lieber-DeCarli ethanol diet was used to expose dams to alcohol throughout the four weeks, an established method which provides a high protein ethanol diet without introducing stress to the mother or compromising the mother’s health^74,75^.

The predicted and validated miRNA-gene target pairs showed miR-29b-3p to be significantly enriched in all groups, targeting a high number of genes downstream. miR-29b-3p was significantly upregulated in all groups with (p < 0.001). This suggests the potential of this miRNA in regulating many gene expressions among both DA and non-DA neurons in the VTA region and the possibility of its involvement in many biological processes across this region in the developmental stages. Recently, it has been shown that miR-29b-3p was downregulated in the PFC of the depressed rats and upregulated by the ketamine treatment. Its overexpression contributed to Ca2 + influx, neuron cell survival, an increase in the extracellular glutamate concentration, and an inhibition in the cell apoptosis. When miR-29b-3p was upregulated, it subsequently improved the depressive behaviors of depressed rats, which could be considered as a potential therapeutic target for treating major depression disorder^76^. Another study showed that miR-29b-3p has been speculated to serve as a potential biomarker for traumatic brain injury with a differential upregulation found in the plasma exosomes^77^. Among the many mRNAs targeted by miR-29b-3p within the alcohol non-DA perinatal exposure group, COL4A1 was one of the validated differentially expressed mRNA targets. Mutations in COL4A1 has been implicated with small vessel disease in the brain and retina^78^ as well as porencephaly which is a rare neurological disease characterized by the existence of degenerative cavities in the brain^79^.

In the nicotine-alcohol DA and alcohol DA perinatal exposure groups, a validated and differentially expressed target of miR-29b-3p was COL18A1. Mutations in this gene have been linked to Knobloch syndrome which is characterized by severe vision impairment as well as skull defects^80,81^. Within the nicotine-alcohol non-DA perinatal exposure group, COL5A2 was a significantly differentially expressed target of miR-29b-3p. Mutations in COL5A2 have been linked to Ehlers-Danlos syndrome which is a group of disorders affecting connective tissues supporting the bones, skin, blood vessels and many organs and tissues throughout the body^82,83^. In the miR-9 genes, the guide strand can be generated from either the 5′ (miR-9-5p) or the 3′ (miR-9-3p), depending on the gene considered^84^. miR-9a-5p was significantly downregulated (p < 0.01) among all exposure groups within the DA and non-DA neurons with a higher significance among the nicotine-alcohol group compared to the alcohol group among both DA and non-DA neurons. Ethanol is a neuroteratogen and disrupts neural maturation and migration during neurogenesis, which suppresses the expression of miR-9^85,86^.This suggests its relationship with the developmental disorders underlying FASD and the teratogen-mediated birth defects underlying perinatal alcohol exposure, an effect that is exacerbated with the combined perinatal exposure with nicotine. miR-9 has been shown to be differentially expressed as an epigenetic inflammation regulator in smokers with lung cancer^87^. miRNA let-7i has been identified as a novel and potent inhibitor of neuronal differentiation^88^. miRNA let-7i was significantly upregulated across all sample groups in the VTA DA and non-DA neurons following both alcohol and nicotine-alcohol exposures. This highlights the importance of let-7i in alcohol exposure during fetal development resulting in a universal expression alteration among different neuron types of the VTA. miR-181a-1-3p was significantly differentially upregulated within the alcohol DA and nicotine-alcohol DA groups. This miRNA, which is suggested to regulate synaptic function and its expression, is induced by dopamine signaling as well as through cocaine and amphetamine exposure in primary neurons^89^. Within the perinatal alcohol and nicotine-alcohol exposure groups in the non-DA VTA neurons, miR-181a-5p was significantly differentially downregulated. This may suggest a neuronal type specific expression modulation due to the drug exposure, in this case alcohol and nicotine. One of the reasons for this could be that the expression of this miRNA is induced by dopamine signaling.

Among the DEG list following perinatal nicotine-alcohol or alcohol exposure in the DA and non-DA neurons, GNAI2, also shown in Table 2, was significantly differentially expressed following both nicotine-alcohol and alcohol perinatal exposures in the DA neurons (p < 0.001) and not significantly expressed in the non-DA neurons. GNAI2 was also significantly differentially expressed in the VTA DA neurons following perinatal alcohol as well as combined nicotine-alcohol exposures in our previous publication. GNAI2 has been suggested to play an important role in the healthy development of the brain as it is involved in axon guidance and cell migration during neuronal development^90^. In vivo studies have shown GNAI2 knockdown mice to exhibit a lack of social interaction, increased anxiety, and long-term depression^91,92^. Among the DEGs following perinatal nicotine-alcohol and alcohol exposure in the non-DA neurons, GRAMD1C, a protein coding gene and associated with Generalized Atherosclerosis^93^, was significantly upregulated (p < 0.001) in both groups, as also shown in Table 2, and significantly downregulated (p < 0.05) in the alcohol DA group. PTOV1 gene was significantly differentially upregulated (p < 0.001) in the DA neurons following both perinatal exposure groups and not significantly expressed in non-DA neurons. Significant overexpression of PTOV1 has been suggested to exhibit an anti-cancer effect when knockdown^94–97^. Synaptogyrin 3 (SYNGR3) was only significantly upregulated in the nicotine-alcohol DA group (p < 0.001). This gene encodes a synaptic vesicle protein that also interacts with the dopamine transporter^98^. Studies suggest that microtubule-associated protein Tau, implicated in Alzheimer’s disease, interacts with SYNGR3 causing synaptic dysfunction. However, lowering the levels of SYNGR3 rescued neurotransmitter release defects induced by presynaptic tau in fly and mouse primary neurons^99,100^. This may suggest an exacerbated effect of combined nicotine and alcohol perinatal exposure on DA neurons and their development along with the possibility of neurodegeneration and early onset of neurodegenerative diseases. Another gene that was only differentially expressed in the perinatal nicotine-alcohol DA exposure group (p < 0.001) was SLC17A6. SLC17A6, also known as VGLUT2, plays a role in L-glutamate transmembrane transporter activity in the glutamatergic pathway and is also co-expressed in DA neurons. SLC17A6/VGLUT2 enables the midbrain DA neurons to co-release glutamate in the NAc and has been highlighted as an emerging player in the complex mechanisms of drug addiction^101,102^. Our previous study showed that SLC17A6 expression was higher (p < 0.05) in the parabrachial pigmented nucleus (PBP) sub-region of the VTA of juvenile rats perinatally treated with nicotine^68^. Our current finding may suggest that perinatal exposure to combined alcohol and nicotine could aggravate nicotine’s effect on SLC17A6 expression in the DA neurons of the VTA and may cause addiction behaviors later in life.

Following our differential expression analysis and miRNA-gene target pairs, our functional enrichment analysis results of DA and non-DA DEGs revealed many GO biological processes and KEGG pathways related to neuronal cell development, proliferation, and survival. The PI3K/AKT pathway is important in regulating the cell cycle progression and apoptosis. Disruption of this pathway leads to decreased cell survival, proliferation, and growth^103–105^. In vitro studies have shown the PI3K signaling pathway to be inhibited after ethanol exposure^105–108^. We have previously indicated that the PI3K/AKT signaling pathway was significantly enriched with miRNA-gene targets in DA neurons but not in non-DA neurons following perinatal nicotine exposure^33^. This study determined many significant DEmiRs-DEGs targets in the DA neurons in the PI3K/AKT signaling pathway compared to fewer DEmiRs and DEGs found in the non-DA neurons following each perinatal exposure group.

Following the nicotine-alcohol perinatal exposure in the DA neurons, significantly downregulated DEmiR (miR-9a-5p) (p < 0.001) was found to inhibit genes within different gene clusters in the PI3K/AKT signaling pathway. KITLG was one of the DEGs targeted by miR-9a-5p following perinatal nicotine-alcohol DA neurons. This gene encodes the ligand of the tyrosine-kinase receptor and plays a role in cell migration^109^. A study on genome-wide analysis of blood DNA methylation levels following childhood trauma in humans showed a locus in the kit ligand gene to have the strongest association with cortisol stress reactivity and early life stress exposure in mice increased both anxiety and KITLG expression in the hippocampus^110,111^. Following the nicotine-alcohol perinatal exposure in the non-DA neurons, significantly downregulated miR-195-5p (p < 0.01) was found to inhibit genes within different clusters in the PI3K/AKT signaling pathway. Emerging studies on miRNAs and their role in disease prognosis and increasing evidence has recently marked miR-195 as a potentially useful tumor biomarker with a significant downregulation in many different cancers^112^. We determined that miR-195 targeted MAPK3, which is involved in cell proliferation, differentiation, and survival^113^. miR-214-3p, which promotes neurogenesis and is highly expressed in neural progenitor cells regulating neocortical development^114^, was significantly downregulated in the PI3K/AKT signaling pathway following perinatal alcohol exposure in the DA neurons (p < 0.05). Among the DEGs targeted by miR-214-3p, PHLPP1 was differentially upregulated (p < 0.001). PHLPP1 has been identified as one of the upregulated candidate genes for down syndrome^115^ which may also show the severe effects of prenatal drug abuse on genetic factors^116^. In the PI3K/AKT signaling pathway following perinatal alcohol exposure on the non-DA neurons, significantly upregulated miR-29b-3p (p < 0.001) was found to target and inhibit PDGFA. PDGFA has been identified as a neurotrophic and synaptic integrity gene, related to neuronal growth and glial changes^117^. Additionally, DEG FGF20 was significantly inhibited by let-7i-5p (p ≤ 0.01) following perinatal alcohol exposure in the non-DA VTA neurons. The fibroblast growth factor (FGF) superfamily of neurotrophic factors are crucial for neural cell development, playing a role in brain assembly and recovery from neural injury^118^. FGF20 plays a role in neurodevelopment, plasticity, and neurodegenerative disorders^118^.

Functional gene enrichment ClueGo analysis among the DA neurons revealed that both perinatal nicotine-alcohol and alcohol exposure groups had neurodegenerative diseases such as Parkinson disease, Huntington disease, and Alzheimer disease to be significantly enriched among the upregulated DEGs. It could be concluded that perinatal exposure to addictive drugs among the DA neurons causes neuronal degeneration, improper neuronal development, oxidative stress, and neuronal death. Ethanol is widely known for its neurotoxic effects on the developing central nervous system, causing apoptotic neurodegeneration in infant rats or mice during synaptogenesis^119^. In the non-DA neurons, perinatal nicotine-alcohol and alcohol exposures showed pathways related to cancer, axon guidance, VEGF signaling pathway, and neurotrophin signaling pathway. All of these pathways play crucial roles in the survival, development, migration, and proliferation of the neurons. In the non-DA perinatal alcohol exposure group, various signaling pathways involved in multiple physiological processes, including cGMP-PKG signaling pathway, G protein-coupled receptor signaling pathway, and Ras signaling pathway, were enriched. These pathways are important in cell development, progression, proliferation, and survival.

Functional enrichment analysis using CluGO on downregulated DEGs following perinatal nicotine-alcohol exposure within the DA neurons showed thermogenesis to be enriched. This pathway has been shown to be impaired following binge-like ethanol exposure during adolescence^120^. Following perinatal nicotine-alcohol exposure in the non-DA neurons, the functional enrichment analysis on the downregulated DEGs showed olfactory transduction and dilated cardiomyopathy (DCM) to be enriched. The toxicity of the ethanol has been determined to affect olfactory receptor genes of fetal mice through maternal binge alcohol consumption, causing odor identification defects and abnormalities in the olfactory system^121^. Following perinatal alcohol exposure in the DA neurons, downregulated DEGs identified signal transduction and cyclic-nucleotide-mediated signaling to be enriched. Neurotoxins and the alterations of cell signaling pathways resulting in functional impairment, cell damage and death have been implied as part of their mechanism following gene expression changes^122^. Following perinatal alcohol exposure in the non-DA neurons, downregulated DEGs recognized olfactory transduction as well as cellular component organization or biogenesis to be enriched. This finding was confirmed by the literature that exosome biogenesis was impacted by ethanol administration in BV-2 cells microglia cell line^123^.

In summary, we identified the miRNome and transcriptome of rat pups following chronic alcohol and nicotine-alcohol exposure during pregnancy. We have analyzed the DEmiRs along with their DEG target pairs, revealing the putative miRNA-gene target interactions following each treatment group for DA and non-DA neurons. We identified altered biological pathways determined by DEmiRs and DEGs in DA and non-DA VTA neurons. We further focused on the enriched PI3K/AKT signaling pathway in each treatment group, identifying its DEmiRs and DEGs. Enriched GO biological processes and KEGG pathways following each treatment group for DA and non-DA neurons were also studied to better understand how addictive substance administration can alter biological processes. Our previous study on perinatal nicotine exposure suggested the ECM-receptor interactions to be significantly altered in DA and non-DA neurons and the PI3K/AKT signaling pathway was enriched in DA neurons, but not in non-DA neurons^33^. These findings showed the importance of DA neurons in neuronal development, apoptosis, and sensitization during developmental stages, suggesting long term effects that may possibly affect the drug addiction pathways. Our study demonstrated that the interaction between miRNA and their predicted mRNA targets can help us to identify and understand their related biological functions. Variable factors including the alcohol intake, nicotine doses and body weight variations across dams are used in this current study. Further investigation is needed to better understand the systemic alterations following perinatal drug exposure in gene network regulation, miRNA-gene target interactions and biological pathways representing specific neuronal subpopulations, and, finally, to investigate therapeutic approaches targeting fetal nicotine-alcohol exposure disorders. Therefore, we are planning to do series of experiments in transgenic animals to further study the interaction between miRNAs and their predicted mRNA targets.

## Materials and methods

### Animal treatment

All experimental protocols and surgical procedures approved by the University of Houston Animal Care Operations (ACO) and the Institutional Animal Care and Use Committee (IACUC) were performed in accordance with accepted guidelines and regulations and carried out in compliance with the ARRIVE guidelines. Pregnant female Sprague–Dawley (SD) rats were purchased from Charles River (Charles River, Wilmington, MA, USA). The animals were housed in the animal facility and maintained at 22 ± 2 °C with 65% humidity on a 12-h light/12-h dark cycle. The animal treatment method has been further detailed^32,33^. Upon arrival, rats were acclimated to the animal facility for 72 h before the subcutaneous osmotic pump (Alzet, Cupertino, CA, USA) was inserted containing either nicotine hydrogen tartrate (Sigma-Aldrich, St. Louis, MO, USA), which released nicotine at a rate of 6 mg/kg/day to simulate the nicotine plasma level found in moderate smokers, or an equal volume of saline for control^30,124^. After the 72-h acclimation time, the pregnant mothers were gradually introduced to a liquid diet containing 36% kcal from ethanol F1265SP or F1264SP control, purchased from Lieber-DeCarli (Bio-Serv, Flemington, NJ, USA) based on the protocol provided by Bio-Serv. This liquid diet model has been well established and used among researchers to reliably produce blood alcohol concentrations (BACs) between 80 and 180 mg/dl in rats, which have been accompanied by neurological deficits similar to what is observed in children with FASD^74,125–131^. On average, pregnant dams consumed about 80–100 ml of liquid diet per day. A total of 12 dams were used; 4 were used for the nicotine-alcohol exposure group, 4 for alcohol exposure group, and 4 for saline (control) group. Pups were exposed to either alcohol alone, combined nicotine and alcohol, or saline for four weeks (gestational day 6 to postnatal day 14), a timeframe that is approximately equivalent to the three trimesters in human pregnancy^19,70–73^. As perinatal drug exposure including but not limited to nicotine, alcohol, and combined exposure has been studied to produce sex dependent changes in the offspring; in this study, only the male pups from each litter were pooled and used^132–135^. Postnatal 10 to postnatal 14-day old male pups were anesthetized using isoflurane gas before decapitation on a VT1200 semiautomatic vibrating blade microtome (Leica, Nussloch, Eisfeld, Germany). 1 mm thick horizontal brain slices containing the VTA were sliced and 1 mm biopsy punch (Integra Miltex, VWR, Radnor, PA, USA) was used to collect the VTA bilaterally. Brain punches from 4 to 7 pups from each litter were pooled and placed on ice in Hibernate A (Gibco, Thermo Fisher Scientific, USA) to preserve and maintain cell viability.

### Brain slice preparations, FACS cell sorting and RNA extraction

Brain slice preparations, FACS cell sorting and RNA extraction were performed as previously described in Keller et al.^33^. The VTA-containing tissue punches were collected and dissociated into a single cell suspension prior to cell sorting using FACS as previously reported by Guez-Barber et al.^136^. Collected tissue punches were briefly dissociated in Accutase (Gibco, Thermo Fisher Scientific, Waltham, MA, USA) and shaken at 4 °C for 30 min. Cells were centrifuged and pelleted at 425×*g* and resuspended in Hibernate A medium (Gibco). Cell aggregates were dissociated through gentle pipetting with increasingly smaller pipette tips. The supernatant which contained the individual cells were then collected. Cellular debris was removed using serial filtration. First, the cell suspension was run through a pre-wetted 100 µm cell strainer and then through a pre-wetted 40 µm cell strainer while on ice. Further removal of small cellular debris was done through density centrifugation. The cell suspension was added to the top of a three-density gradient that was made using Percoll (GEHealthcare, VWR, USA) and centrifuged at 430×*g* for 3 min. After centrifugation, the cloudy top layer containing cellular debris was removed. The remaining cell suspension was pelleted through centrifugation at 550×*g* for 5 min.

For immunolabeling, cells were fixed following resuspension in equal parts of Hibernated A and 100% cold ethanol, gentle vortexing, and kept on ice for 15 min. Cells were incubated and labelled with conjugated primary antibodies neuronal marker, NeuN/Alexa Fluor 488 (NeuN/AF488, ab190195, Abcam, Cambridge, MA, USA), and tyrosine hydroxylase/phycoerythrin (TH/PE, ab209921, Abcam) and rotated for 30 min at 4 °C. Cells were washed with PBS and centrifuged at 950×*g* for 3 min before they were resuspended in PBS. Flow cytometry was performed on an (LSR II) FACS Aria (BD Biosciences, Franklin Lakes, NJ, USA) instrument and analyzed using FlowJo software at the Baylor College of Medicine Cytometry and Cell Sorting Core (One Baylor Plaza, Houston, TX, USA). Neurons with intact nuclei were labelled and sorted. NeuN + /TH− population of cells were labeled non-DA neurons and double stained, NeuN + /TH + cell populations were labeled DA neurons, and all were collected. Populations were distinguished by their forward and side scatter, and two-parameter density plots were measured with gating parameters set at around 10^3^ for NeuN-FITC and 10^4^ for TH-PE expression. Following FACS, cells were formed into a pellet through centrifugation at 2650×*g* for 8 min at 18 °C and total RNA was extracted using miRNeasy Micro Kit (Qiagen, Hilden, Germany) including DNAse treatment following manufacturer’s instructions. A NanoDrop 2000 spectrophotometer (Thermo Fisher Scientific) was used to check the RNA purity and quantity according to the optical density (OD) of each sample at 260 nm and 280 nm. Only samples with a 260/280 ratio of 1.9 or greater were used in experiments.

### Microarray preparation, labeling, and hybridization for gene and microRNA expression profiling

Microarray kits for mRNA and miRNA expression analysis were purchased from Agilent (Santa Clara, CA, USA) as previously described in Keller et al.^33^. mRNA expression profiling was done using SurePrint G3 Rat Gene Expression v2 8 × 60 K microarray (ID: 074036) with 30,584 unique gene probes using 25 ng of total RNA. Samples were prepared and labeled according to manufacturer’s instructions for the One-Color Microarray-Based Gene Expression Analysis using the One-Color Low Input Quick Amp Labeling kit with RNA Spike-Ins. RNeasy Mini Kit (Qiagen) was used on the labeled amplified complimentary RNA which was then quantified using a NanoDrop 2000 spectrophotometer, and cRNA yield and specificity was calculated according to manufacturer’s instructions. Microarrays were hybridized for 17 h at 65℃ using the Gene Expression Hybridization kit according to manufacturer’s instructions. miRNA expression profiling was prepared using an 8 × 15 K Rat miRNA Microarray, Release 21.0 (ID: 070154) containing 758 mature miRNAs using a starting quantity of 100 ng total RNA containing miRNAs following manufacturer’s instructions of the MicroRNA Microarray System with miRNA Complete Labeling and Hyb Kit. Micro Bio-Spin P-6 gel column (Bio-Rad, Hercules, CA, USA) was used for the purification of the labeled RNA following manufacturer’s instructions. Samples were dried using a vacuum concentrator with heater at 50℃ and hybridized at 55 °C for 20 h. All slides were washed using Gene Expression Wash Buffers containing Triton X-102 following manufacturer’s instructions and scanned using a G4900DA SureScan Microarray Scanner. Feature Extraction Software v12.0.1 was used to collect the raw microarray data from the resulting images.

### Data analysis

All genomic data analysis including pre-processing, normalization, and statistical analyses were performed using Bioconductor packages in R version 3.6.1^137^. The limma^138^ package was used to import and analyze mRNA microarray data and for the removal of outliers, background correction, and quantile normalization using the “normexp” method. Low expression was defined through intensity of less than 75% brighter than 90% intensity of negative controls. The lmFit function was used to calculate fold change and standard errors, which fits multiple linear models by weighted least squares. Standard errors were moderated using the eBayes function, which computes log-odds of differential expression using an empirical Bayes model. DEGs were identified using a series of p values (i.e. 0.05, 0.01, 0.001), with the minimum log2 fold change > 1 and adjusted using the BH method.

To analyze the miRNA microarrays, raw intensity data was imported and the AgiMicroRna^139^ package was used for processing. Raw data was loaded using the readMicroRnaAFE function and preprocessed using the rmaMicroRna function, which implements the robust multi-array average (RMA) algorithm. The data was then filtered using the filterMicroRna function, and genes were detected only if they were expressed in at least 50% of samples, with higher intensity than the mean value of negative control + 1.5 standard deviations, which were then picked for analysis. The linear model was fitted to the miRNA expression data and moderated statistics were calculated using eBayes. Differential expression was identified using a series of p values (i.e. 0.05, 0.01, 0.001), with the minimum log2 fold change > 0.5 and adjusted using the BH method.

### Integrated analysis of differentially expressed miRNA-mRNA target pairs and functional enrichment analysis

MultiMiR^59^ package was used to identify the predicted and validated miRNA-mRNA pairs based on the inverse correlation regulation between miRNA and target genes. Probability of significance was assessed using a pair-wise Pearson correlation analysis on each miRNA-mRNA predicted target pairs. miRNA-gene target pairs with parameters of r < − 0.5 and q < 0.05 were used for the miRNA-gene correlation networks^140^. Functional enrichment analysis was performed on the DEG lists using DAVID version 6.8^60,61^ and ClueGO v2.5.6^62^ plugin for Cytoscape v3.8^63^. Gene ontology (GO) terms and Kyoto Encyclopedia of Genes and Genomes (KEGG) pathways were both included in the ClueGO analysis. ClueGO performed single cluster analysi(s and comparison of clusters to create a network of functionally related terms that reflects the relationship between the terms biological processes using the DEG lists. Statistical significance was performed and p-values were calculated using a two-sided hypergeometric test and corrected for multiple testing using BH method.

### Quantitative RT-qPCR validation of microarray data

The microarray results were validated using RT-qPCR (Supplementary Fig. S9) as described elsewhere^6,7^. All reagents and kits used for quantitative reverse transcription polymerase chain reaction (RT-qPCR) were purchased from Applied Biosystems (Thermo Fisher Scientific, Carlsbad, CA, USA) unless stated otherwise. Total RNA was isolated using RNeasy Mini Kit (Qiagen, Hilden, Germany) according to manufacturer’s instructions. cDNA was prepared using High Capacity cDNA Reverse Transcription Kit (Applied Biosystems, Thermo Fisher Scientific) according to manufacturer’s instructions and reverse transcription (RT) was performed on a T100 thermal cycler (Bio-Rad, Hercules, CA, USA). All primers used for all reactions were TaqMan Gene Expression Assays (Thermo Fisher Scientific). Lypla2 (Assay ID: Rn00580197_m1), Bnip3l (Assay ID: Rn01534668_g1), Gnai2 (Assay ID: Rn01447850_m1), Gtf2i (Assay ID: Rn01499727_m1), Ndrg2 (Assay ID: Rn01414698_m1), Atp2a2 (Assay ID: Rn00568762_m1), Icmt (Assay ID: Rn01516590_m1), Ank1 (Assay ID: Rn01756750_m1), and Gramd1c (Assay ID: Rn01475288_m1). Comparative Ct method (ΔΔCt) was calculated using StepOnePlus Real-Time PCR System and used to determine relative expression values. Triplicate RT-qPCR reactions were performed in all validation experiments. Real-time PCR was carried out using TaqMan Fast Advanced Master Mix purchased from Applied Biosystems (Thermo Fisher Scientific) and corresponding TaqMan Assays on a StepOnePlus Real-Time PCR System (Applied Biosystems, Thermo Fisher Scientific) according to manufacturer’s instructions using the following parameters: 2 min at 50 °C, 2 min at 95 °C, 40 cycles at 1 s at 95 °C and 20 s at 60 °C. All reactions were performed in triplicate. Significance was computed using Student’s t-test with BH-correction for multiple testing with false discovery rate of 0.05.

## Supplementary Information

Supplementary Information

## Data Availability

The datasets generated during and/or analyzed during the current study are available from the corresponding author upon reasonable request.
