# Supplementary material for: Investigating the effects of chronic perinatal alcohol and combined nicotine and alcohol exposure on dopaminergic and non-dopaminergic neurons in the VTA
Source: Sci Rep. 2021 Apr 22;11:8706. doi: 10.1038/s41598-021-88221-8 (PMC8062589; doi:10.1038/s41598-021-88221-8)
Supplement: Supplementary file 1 — Supplementary Information [file 41598_2021_88221_MOESM1_ESM.pdf]

Investigating the effects of chronic perinatal alcohol and combined nicotine and alcohol exposure on dopaminergic and non-dopaminergic neurons in the VTA

Tina Kazemi1, Shuyan Huang1, Naze G. Avci1, Yasemin M. Akay1, and Metin Akay1*

University of Houston, Department of Biomedical Engineering, Houston, TX, 77204, USA
*Corresponding author: makay@uh.edu

Supplementary Table S1. Enriched KEGG pathways. KEGG pathways enriched following perinatal (a) nicotine-alcohol exposure on DA, (b) nicotine-alcohol exposure on non-DA, (c) alcohol exposure on DA, and (d) alcohol exposure on non-DA along with the corresponding DEGs identified within each KEGG pathway for the upregulated and downregulated DEG lists.

(a)

**Perinatal Nicotine-Alcohol Exposure (DA)**					
**KEGG Term**	**P value**	**Genes**	**KEGG Term**	**P value**	**Genes**
**Upregulated**			**Downregulated**		
Ribosome	2.68E-38	RPL18, RPL17, RPL36A, RPL19, RPL14, RPL13, RPLP2, RPS2, RGD1564062, RPS3, RGD1561736, RPS3A, RPLP1, RPL10, FAU, RPL11, RPL12, RPS27A, RPL36AL, RGD1562415, RPL35A, RGD1563459, LOC681260, RPS4X, RGD1564744, RPS18, RPS19, RPL41, RPS16, RPS17, RPS15, RPS12, RPS13, RPS11, UBA52, LOC500371, RPL35, RPL27A, RPL36, RPS15A, RPL37, RPL38, RPL39, RPS25, RPS26, RPL30, RPS27, RPS29, RPL32, RPL7, RPL31, RPL6, RPL34, RPL9, RPL37A-PS1, RPL8, RPL7A, RPS20, RPL4, RPL10A, RPS21, RGD1560633, RPL26, RPS9, RPL24, RPL23A, RPS6, RPS5, RPS8, RPL23, RPL18A, RPL22, RPL13A, RPL21, RPL37A	Olfactory transduction	6.31E-03	OLR1156, OLR1151, OLR1091, OLR23, OLR1349, CNGB1, OLR463, OLR466, OLR655, OLR1200, CLCA2, OLR588, OLR1165, OLR1600, OLR1601, OLR35, OLR1605, OLR702, OLR705, OLR251, OLR646, OLR1684, OLR789, OLR103, OLR592, OLR1742, OLR105, OLR1749, OLR1365, OLR1468, LOC682961, OLR44, OLR677, OLR1557, OLR486, OLR485, OLR285, OLR302, OLR143, OLR282, OLR1654, OLR1458, OLR321, OLR881, OLR1455, OLR1454, OLR1147, OLR877, OLR767, OLR1563, OLR1767, OLR68, OLR1306, OLR889, OLR1307, OLR418, OLR62, OLR234, OLR378, OLR329, LOC687881, OLR1102, OLR1249, OLR1393, OLR327, OLR1394, OLR1252, OLR1644, OLR1643, OLR1063, OLR404, OLR604, OLR1504, OLR550, OLR1701, OLR1585, OLR410, OLR192, OLR1325, OLR496, OLR1075, OLR499, OLR1514, OLR1425, OLR1179, OLR1228, OLR1699, OLR1122, PDE1C, OLR1071, OLR440, OLR1607, OLR206, OLR1609, OLR425, OLR429, OLR721, OLR1523, OLR1186, OLR1522, OLR360, OLR527, OLR1413, OLR1387, OLR1409
Parkinson's disease	2.26E-11	UQCRC2, ATP5D, UCHL1, CYC1, PINK1, COX5B, NDUFS7, NDUFS6, NDUFS5, NDUFS4, RGD1566212, ATP5H, ATP5J, NDUFA10L1,	Basal cell carcinoma	9.36E-03	FZD8, DVL3, WNT1, BMP2, WNT4, WNT5B, FZD3, WNT9A, GLI2, GLI3, CTNNB1

		NDUFB10, SLC25A4, SLC25A5, UBE2J1, NDUFC2, COX4I1, NDUFA10, COX6C, UQCRH, LOC684509, UBC, UBB, NDUFB3, NDUFB4, LOC680288, NDUFB7, NDUFB8, NDUFB9, COX7B, TH, ATP5G2, ATP5G1, COX7A2L, COX6B2, NDUFA4, NDUFA5, COX7A2, NDUFA8, NDUFA6, NDUFA7, COX8A, VDAC2, VDAC3, NDUFA1, PARK7, UBA1, PPID, NDUFV1, SDHD, SLC18A2, COX6A1, ATP5A1			
Huntington's disease	2.35E-10	ATP5D, UQCRC2, CLTA, CLTB, AP2S1, CYC1, REST, CLTC, COX5B, NDUFS7, NDUFS6, GPX1, NDUFS5, NDUFS4, RGD1566212, DLG4, CREB3L2, ATP5H, TBPL1, ATP5J, NDUFA10L1, NDUFB10, SLC25A4, SLC25A5, NDUFC2, COX4I1, NDUFA10, COX6C, UQCRH, LOC684509, NDUFB3, NDUFB4, POLR2G, LOC680288, POLR2E, NDUFB7, POLR2L, NDUFB8, NDUFB9, COX7B, ATP5G2, COX7A2L, ATP5G1, POLR2B, COX6B2, HAP1, AP2M1, NDUFA4, NDUFA5, COX7A2, NDUFA8, NDUFA6, COX8A, NDUFA7, GRIN1, SOD1, VDAC2, VDAC3, NDUFA1, PPID, NDUFV1, SDHD, COX6A1, ATP5A1	Cytokine-cytokine receptor interaction	1.48E-02	IL1R2, IL22RA1, CXCL9, FASLG, IL13, CD70, IL15, CXCL12, CCL4, IL10, CXCL10, IL12RB2, CCL25, CXCR4, IFNA4, IFNK, LTA, CD27, EPO, IL4, IL21, LEP, TNFRSF9, CCR6, NGFR, MPL, IL2
Oxidative phosphorylation	8.57E-09	UQCRC2, ATP5D, ATP6AP1, CYC1, COX5B, NDUFS7, NDUFS6, NDUFS5, NDUFS4, RGD1566212, ATP5L, ATP5I, COX17, ATP5H, ATP5J, NDUFA10L1, NDUFB10, NDUFC2, COX4I1, NDUFA10, COX6C, UQCRH, LOC684509, NDUFB3, NDUFB4, LOC680288, NDUFB7, NDUFB8, NDUFB9, COX7B, ATP5G2, ATP5G1, COX7A2L, ATP6V0C, COX6B2, NDUFA4, TCIRG1, NDUFA5, COX7A2, NDUFA8, NDUFA6, COX8A, NDUFA7, LHPP, NDUFA1, NDUFV1, SDHD, COX6A1, ATP5A1	p53 signaling pathway	2.22E-02	CCNE2, BID, RPRM, CDK6, SFN, FAS, RCHY1, XRN1, GTSE1
Alzheimer's disease	6.46E-08	ATP5D, UQCRC2, CYC1, IDE, COX5B, NDUFS7, NDUFS6, NDUFS5, APP, NDUFS4, LOC303448, RGD1566212,	Intestinal immune network for IgA production	3.32E-02	IL4, CCL25, CXCR4, IL15, RT1-DOA, CXCL12, ICOSLG, IL10, IL2

		APOE, GRIN2C, MAPT, PSENEN, ATP5H, ATP5J, NDUFA10L1, NDUFB10, NDUFC2, COX4I1, NDUFA10, COX6C, MAPK1, PSEN1, UQCRH, LOC684509, PSEN2, NDUFB3, NDUFB4, LOC680288, NDUFB7, NDUFB8, NDUFB9, COX7B, PPP3R1, ATP5G2, COX7A2L, ATP5G1, PPP3CC, COX6B2, PPP3CA, GAPDH, NDUFA4, LPL, NDUFA5, COX7A2, NDUFA8, NDUFA6, NDUFA7, COX8A, GRIN1, NDUFA1, NCSTN, ATP2A2, NDUFV1, GSK3B, SDHD, COX6A1, ATP5A1			
Endocytosis	2.17E-05	CLTA, CLTB, CHMP5, AP2S1, VPS37A, VPS37B, VPS37C, CLTC, CDC42, SMAP2, ATG2A, GIT2, AGAP2, RT1-T24-4, AGAP3, EGFR, EPN3, PLD1, PSD2, RT1-EC2, RT1.AA, RT1-A2, RT1-A1, CHMP1A, ARRB2, ACAP3, PSD, ARRB1, ACAP2, PDGFRA, ARFGAP1, FGFR2, PARD3, FGFR3, RT1-M3-1, ADRBK2, SRC, CHMP2B, SH3GLB1, RPL9, RT1-M6-2, HSPA8, AP2M1, CSF1R, STAMBP, DNM3, RT1-CE1, TGFBR1, KDR, RAB31, HGS, DNM2, RT1-N2	Keratan sulfate biosynthesis	4.16E-02	ST3GAL3, ST3GAL2, CHST4, B3GNT2
Neurotrophin signaling pathway	2.65E-04	IRAK2, YWHAZ, CAMK2G, FASLG, BAD, MAPK10, RPS6KA3, ATF4, YWHAH, BAX, SOS1, NTRK2, MAPK3, RAC1, CAMK2D, RAP1B, SHC1, RHOC, IKBKB, SHC2, PIK3R1, ARHGDIB, YWHAZ, CAMK2G, FOXO3, CDC42, MAP3K1, SOS2, SH2B3, PIK3CA, NGFRAP1, CSK, MAP2K7, ARHGDIA, AKT2, IRAK1, YWHAB, KIDINS220, YWHAE, NTRK3, RPS6KA5, MAPK1, YWHAG, RPS6KA1, PSEN1, GSK3B, MAPK14, NTRK2, YWHAQ, MAPK9	Notch signaling pathway	5.18E-02	HES1, DVL3, NOTCH1, CIR1, APH1A, DTX3L, MAML1, DTX3, RBPJL
Lysosome	3.96E-04	CLTA, CLTB, AP4E1, ATP6AP1, ACP2, CTSA, ABCA2, CLTC, SLC11A2, ATP6V0C, LAPTM5, GNPTAB, IDS, MAN2B1, GBA, TCIRG1, LIPA, CTSS, CD63, CD164, M6PR, FUCA1, MANBA, LAMP1, SLC17A5, LAMP2,	Calcium signaling pathway	5.56E-02	TRPC1, GNA14, CYSLTR1, TNNC1, TACR1, MYLK3, GRIN1, NTSR1, CACNA1S, P2RX5, P2RX6, HRH2, PDE1C, AVPR1B, PDE1A, PLCG2, RYR1, ADRA1A, RYR2, NOS3, CACNA1C, F2R, HTR2A

		IGF2R, SMPD1, CTSE, CTSD, CTSB, CTSH			
Antigen processing and presentation	1.13E-03	HSP90AB1, RT1-M3-1, PDIA3, IFI30, RT1-DMB, CALR, CANX, B2M, LOC499644, LOC679973, HSPA4, HSPA5, RT1-M6-2, RT1-T24-4, HSPA8, HSP90AA1, RT1-CE1, RFX5, CTSS, RT1-EC2, RT1.AA, RT1-A2, RT1-A1, PSME2, CTSB, WDR46, RT1-N2	Ubiquitin mediated proteolysis	6.91E-02	UBE2E3, WWP2, LOC691764, WWP1, CDC23, UBA6, CDC16, ANAPC7, RCHY1, ITCH, UBE2D1, BRCA1
TGF-beta signaling pathway	1.22E-03	PPP2R1B, PPP2R1A, TGFBR2, CREBBP, BMPR2, SMAD3, SMAD2, TGFB2, ACVR2B, EP300, ID2, ID1, ZFYVE16, MAPK3, RHOC, BMPR1A	Hedgehog signaling pathway	6.94E-02	WNT1, BMP2, WNT4, WNT5B, WNT9A, GLI2, GLI3, ZIC2, BMP6, WNT5A, SMO, CSNK1G1, LRP2, SUFU, WNT2B, BMP8A
Cardiac muscle contraction	2.68E-03	UQCRC2, CACNA2D1, ATP1B1, COX7A2, ATP1B3, ATP1B2, ACTA2, COX8A, COX7B, CYC1, COX4I1, CACNB3, ATP1A2, COX7A2L, CACNA2D2, COX5B, COX6C, TPM3, ATP2A2, UQCRH, COX6A1, COX6B2	Complement and coagulation cascades	7.56E-02	C3AR1, FGG, KNG2, FGA, C1S, C8G, RGD1559810, PLAUR
Long-term potentiation	2.93E-03	ADCY1, CAMK2G, CREBBP, GRIN2A, PRKCB, PLCB3, RPS6KA3, ATF4, EP300, MAPK3, CAMK2D, RAP1B, PRKACB, PRKCA, CAMK2G, GRIN1, PPP3R1, PPP1CC, PRKX, PRKCB, MAPK1, PPP1CA, RPS6KA1, GRIA1, GRIN2C, PPP1R1A, PPP3CC, PRKACA, PPP3CA	Regulation of autophagy	8.31E-02	ATG4C, ATG4B, IFNA4, ULK3, PRKAA2, PIK3R4
Colorectal cancer	5.36E-03	TGFBR2, CYCS, SMAD3, SMAD2, BAD, MAPK10, TGFB2, CCND1, SOS1, BAX, RAC1, MAPK3, PIK3R1, AXIN1	TGF-beta signaling pathway	8.38E-02	INHBA, ACVR1B, LTBP1, INHBC, TGFBR2, PITX2, ACVR1, BMP8A, THBS4
mTOR signaling pathway	6.35E-03	STRADA, RPS6KB2, RPS6, EIF4B, MAPK1, PDPK1, HIF1A, RPS6KA1, TSC1, ULK1, TSC2, VEGFA, PIK3CA, RHEB, MLST8, AKT2			
Lysine degradation	8.47E-03	DLST, TMLHE, ALDH2, SETD7, WHSC1, ACAT2, NSD1, HADHA, SUV39H2			

Wnt signaling pathway	8.68E-03	PPP2R1B, PPP2R1A, CAMK2G, CREBBP, SMAD3, SMAD2, MAPK10, PRKCB, CTNNBIP1, MAP3K7, PLCB3, CCND1, EP300, SFRP2, RAC1, CAMK2D, RHOC, PRKACB, WNT7A, AXIN1	
Pancreatic cancer	1.06E-02	VEGFC, CCND1, TGFBR2, MAPK3, RAC1, SMAD3, SMAD2, MAPK10, BAD, IKBKB, PIK3R1, TGFB2	
Ubiquitin mediated proteolysis	1.12E-02	SAE1, CDC34, ANAPC11, STUB1, RBX1, CUL2, UBE2D3, CUL5, MGRN1, MAP3K1, TRIP12, LOC689226, ANAPC5, VHL, ANAPC4, UBE2J1, CDC23, HERC4, UBE2I, SKP1, UBOX5, CDC26, UBE2B, RFWD2, PIAS4, UBA1, UBE2K, UBA3, TCEB2, UBE4A, UBE2G1, UBE2I, HERC2, UBE2Q2, UBE2L3, UBE2Q2L, ERCC8, CUL5, UBE2D2, NEDD4, KLHL9, UBE2M, PIAS2, TCEB1	
Nucleotide excision repair	1.13E-02	RFC5, ERCC8, XPC, ERCC6, POLD1, POLD2, GTF2H3, ERCC3, GTF2H2	
Chemokine signaling pathway	1.23E-02	PARD3, ADCY1, BCAR1, GNG12, PXN, PRKCB, CCR9, GNG8, PLCB3, ADCY9, GNB1, SOS1, MAPK3, RAC1, RAP1B, RHOC, SHC1, PRKACB, IKBKB, GNG5, SHC2, PIK3R1	
Fatty acid metabolism	1.29E-02	GCDH, ACADSB, ACSL1, ACADS, ADH5, HADH, ACADL, ACSL3, ACAT1, ACSL6, HADHA, ALDH9A1, ACOX3	
Spliceosome	1.41E-02	DHX8, NCBP1, NHP2L1, TRA2A, SNRPD1, SNRPC-PS1, SF3B5, HNRNPA1, RBMX, HNRNPA3, HNRNPK, DHX38, DDX23, PRPF8, USP39, SNRPG, DDX42, NHP2L1, SMNDC1, SF3B1, PCBP1, DHX15, LSM5, PQBP1, LSM3, SNRNP70, ACIN1, HSPA8, RBM25, SNRPN, CDC5L, SF3A2, DDX5, PRPF18, RBMX, PRPF4, HNRNPU, SF3A3, LOC363208, EIF4A3, AQR, SNRPB, PRPF38A	
Pentose phosphate pathway	1.57E-02	ALDOA, GPI, RPE, ALDOC, PFKP, TKT, PFKM, PRPS2, PRPS1	

Adherens junction	1.75E-02	MAP3K7, PARD3, EP300, BAIAP2, PVRL3, TGFBR2, MAPK3, RAC1, CREBBP, SMAD3, RHOC, SMAD2, ACTB, EGFR, PTPN6, FGFR1, PARD3, PTPRF, ACTN4, BAIAP2, TGFBR1, NLK, WASF2, SRC, ACTG1, CDC42, MAPK1, PTPN1, SSX2IP	
MAPK signaling pathway	1.78E-02	MKNK2, CACNB1, FASLG, GNG12, TGFB2, MAP3K7, TNFRSF1A, PLA2G12A, SOS1, RAC1, PRKACB, RASA1, CACNA2D1, TAOK2, PTPN5, TGFBR2, MAPK10, ECSIT, PRKCB, RPS6KA3, ATF4, MAPK3, NTRK2, CACNA1G, RAP1B, IKBKB, PLA2G3, DUSP8, MAP3K12, CACNA1B, PDGFA, GNA12, PRKX, CDC42, MAPT, PRKACA, FGF1, MAP2K7, AKT2, PRKCA, EGFR, PTPRR, DDIT3, PRKCB, MAP4K3, MAP4K4, MAPK1, ARRB2, RASGRF1, ARRB1, PDGFRA, MAPK9, STMN1, FGFR2, FGFR1, FGFR3, MRAS, MAPKAPK3, PPP3R1, CACNB3, GNG12, SRF, MAP3K1, SOS2, PPP3CC, PPP3CA, NFATC2, HSPA8, CACNA2D1, TGFBR1, NLK, CACNA2D2, RPS6KA5, DUSP3, RPS6KA1, MAPK14, NTRK2, MAPK8IP2, MAPK8IP3, MAPK8IP1, DUSP6	
Proteasome	2.19E-02	SHFM1, PSMA7, PSMB4, PSMB6, PSMC5, PSMA6, PSMD11, PSME2, PSMA4, PSMC2, PSMD1, PSMD4, PSME4, PSMD6, PSMB10, PSMB5, PSMA2, PSMC6, PSME1, PSMA3L, PSMA7, PSMD7	
Axon guidance	2.21E-02	ABLIM2, GNAI2, PPP3R1, L1CAM, LRRC4C, ITGB1, CDC42, PPP3CC, PPP3CA, NFATC2, ROCK1, PLXNB1, DPYSL2, ARHGEF12, SLIT1, EPHA5, MAPK1, SEMA6B, EPHA6, SEMA6D, EPHA8, RGS3, GSK3B, SEMA4B, SEMA4D, SRGAP1, SEMA4A, SRGAP2	

Fc gamma R-mediated phagocytosis	2.32E-02	PRKCA, DNM3, PLD1, LYN, MARCKSL1, WASF2, RPS6KB2, ARPC5, VASP, PRKCB, CDC42, ARPC1B, MAPK1, ARPC3, GSN, FCGR1A, PIK3CA, FCGR2A, PPAP2B, AKT2, DNM2	
Focal adhesion	2.60E-02	TLN1, BCAR1, ITGA1, IGF1, BAD, MAPK10, COL5A1, PXN, PRKCB, VEGFC, CCND1, ARHGAP5, SOS1, MAPK3, RAC1, RELN, RAP1B, RHOC, SHC1, COL11A2, SHC2, PIK3R1, MYLK	
Renal cell carcinoma	2.69E-02	VEGFC, EP300, SOS1, MAPK3, RAC1, CREBBP, RAP1B, TCEB1, PIK3R1, TGFB2, ARNT	
Glioma	3.14E-02	CCND1, SOS1, CAMK2G, MAPK3, CAMK2D, IGF1, SHC1, SHC2, PIK3R1, PRKCB	
Amino sugar and nucleotide sugar metabolism	3.43E-02	CYB5R3, GMPPB, CYB5R1, GNE, UGDH, GALT, NPL, PMM2	
Cysteine and methionine metabolism	3.88E-02	DNMT3A, GOT1, RGD1560523, BHMT, RGD1562690, ENOPH1, APIP	
GnRH signaling pathway	3.96E-02	ADCY1, CAMK2G, MAPK10, PRKCB, PLCB3, ATF4, ADCY9, PLA2G12A, SOS1, MAPK3, CAMK2D, PRKACB, PLA2G3	
Chronic myeloid leukemia	4.46E-02	CCND1, SOS1, TGFBR2, MAPK3, SMAD3, SHC1, BAD, IKBKB, SHC2, PIK3R1, TGFB2	
Valine, leucine and isoleucine degradation	4.73E-02	MUT, ACADSB, BCAT2, IVD, BCKDHB, ALDH2, ACAT2, HADHA	
Amyotrophic lateral sclerosis (ALS)	6.30E-02	ALS2, TNFRSF1A, BAX, RAC1, CYCS, GRIN2A, CCS, APAF1, BAD	
Gap junction	6.73E-02	PRKCA, EGFR, GNAI2, TUBB2B, PDGFA, TUBB2A, TUBA3B, GJA1, PRKX, SRC, PRKCB, MAPK1, CSNK1D, SOS2, TUBB5, PDGFRA, PRKACA, GNAS, TUBA1A, TUBA1B	

Melanogenesis	7.01E-02	PLCB3, ADCY1, EP300, ADCY9, CREB1, CAMK2G, MAPK3, CREBBP, CAMK2D, PRKACB, WNT7A, PRKCB	
Regulation of actin cytoskeleton	7.27E-02	FGFR2, FGFR1, FGFR3, APC2, PDGFA, MRAS, SSH3, GNA12, WASF2, ABI2, GNG12, ARPC5, ITGB1, ACTG1, CDC42, PFN1, PFN2, EZR, ARPC3, GSN, ITGAV, SOS2, PIK3CA, FGF1, CSK, ACTB, EGFR, ARHGEF1, ROCK1, ACTN4, BAIAP2, ARHGEF12, MYH9, PPP1CC, ARPC1B, MAPK1, PPP1CA, PDGFRA, TMSB4X	
Fructose and mannose metabolism	7.92E-02	ALDOA, KHK, PFKFB4, PFKFB3, ALDOC, PFKP, PFKM, MTMR7, PMM1	
Notch signaling pathway	8.55E-02	NCSTN, DTX4, CTBP1, CTBP2, PSEN1, PSEN2, JAG2, PSENEN, RBPJ, LFNG, NCOR2, DVL1	
Steroid biosynthesis	8.59E-02	CYP51, TM7SF2, SOAT1, LIPA, SQLE, DHCR7	
Propanoate metabolism	8.86E-02	MUT, MLYCD, ALDH2, RGD1562690, ACAT2, HADHA	
Apoptosis	8.90E-02	IRAK2, TNFRSF1A, BAX, CYCS, FASLG, APAF1, PRKACB, BAD, IKBKB, IL3RA, PIK3R1	
N-Glycan biosynthesis	9.19E-02	GCS1, MAN2A2, B4GALT3, MGAT5B, STT3A, RPN1, MAN1B1, DPM2, DPM3, RPN2, ALG11, MAN2A1, MGAT3, GANAB, B4GALT2, ALG3, MAN1A1, DDOST	
Glycerolipid metabolism	9.19E-02	DGKA, LPL, DGAT1, DGKB, AKR1A1, MGLL, AGPAT4, PPAP2B, AGPAT3, ALDH9A1, AGPAT1	
Basal transcription factors	9.80E-02	TAF13, TAF9B, GTF2H3, TAF9, GTF2B, GTF2H2	

(b)

Perinatal Nicotine-Alcohol Exposure (non-DA)		
**KEGG Term**	**P value**	**Genes**
**Upregulated**		
Neurotrophin signaling pathway	8.71E-03	MAPK1, PSEN1, CAMK2G, MAPK3, GAB1, RAC1, FASLG, PIK3R1
Renal cell carcinoma	8.86E-03	MAPK1, PDGFA, MAPK3, GAB1, RAC1, PIK3R1
Axon guidance	9.08E-03	MAPK1, PTK2, EFNB2, MAPK3, RAC1, PPP3R1, SEMA4D, SEMA4A
Focal adhesion	1.00E-02	ACTB, MAPK1, PTK2, PDGFA, TNR, MAPK3, RAC1, COL1A2, PIP5K1B, PIK3R1
VEGF signaling pathway	1.18E-02	MAPK1, PTK2, MAPK3, RAC1, PPP3R1, PIK3R1
T cell receptor signaling pathway	1.55E-02	MAPK1, MAPK3, PPP3R1, CD4, PIK3R1, DLG1, CD28
ErbB signaling pathway	2.05E-02	MAPK1, PTK2, CAMK2G, MAPK3, GAB1, PIK3R1
Viral myocarditis	2.24E-02	ACTB, EIF4G2, RT1-CE1, RAC1, CD28, RT1-N2
Prostate cancer	2.56E-02	CCNE2, MAPK1, PDGFA, MAPK3, FOXO1, PIK3R1
Autoimmune thyroid disease	2.62E-02	RT1-CE1, FASLG, TSHR, CD28, RT1-N2
Glioma	2.62E-02	MAPK1, PDGFA, CAMK2G, MAPK3, PIK3R1
Pathways in cancer	3.26E-02	CCNE2, MAPK1, PTK2, PDGFA, MAPK3, RAC1, MITF, FASLG, FOXO1, PTCH1, PIK3R1, CSF1R
Natural killer cell mediated cytotoxicity	3.79E-02	MAPK1, MAPK3, RAC1, PPP3R1, FASLG, PIK3R1
Melanoma	3.89E-02	MAPK1, PDGFA, MAPK3, MITF, PIK3R1
Regulation of actin cytoskeleton	4.00E-02	ACTB, MAPK1, PTK2, PDGFA, MAPK3, RAC1, PIP5K1B, CYFIP1, PIK3R1
B cell receptor signaling pathway	5.03E-02	MAPK1, MAPK3, RAC1, PPP3R1, PIK3R1
Cell adhesion molecules (CAMs)	5.68E-02	RT1-CE1, CNTN2, CNTN1, CD4, CD28, RT1-N2, CLDN15
Graft-versus-host disease	6.86E-02	RT1-CE1, FASLG, CD28, RT1-N2
Spliceosome	7.41E-02	HNRNPA3, SF3B1, HNRNPK, DHX15, DDX5, PRPF40A
Allograft rejection	7.51E-02	RT1-CE1, FASLG, CD28, RT1-N2
Fc gamma R-mediated phagocytosis	8.06E-02	MAPK1, MAPK3, RAC1, PIP5K1B, PIK3R1
Antigen processing and presentation	8.06E-02	RT1-CE1, CD4, CTSS, CALR, RT1-N2
Toll-like receptor signaling pathway	8.60E-02	MAPK1, MAPK3, RAC1, TICAM2, PIK3R1
Chemokine signaling pathway	9.82E-02	MAPK1, PTK2, MAPK3, RAC1, GNB4, GRK5, PIK3R1
**Downregulated**		
Arrhythmogenic right ventricular cardiomyopathy (ARVC)	4.39E-03	PKP2, LMNA, LEF1, ITGA3, CACNG2, CTNNA1

Dilated cardiomyopathy	9.98E-03	LMNA, GNAS, ITGA3, CACNG2, TPM2, TPM3
GnRH signaling pathway	1.19E-02	MAP2K1, CALML3, GNAS, MMP14, SRC, PLA2G4E
Ether lipid metabolism	1.31E-02	PAFAH2, PLA2G7, PLA2G4E, AGPAT1
Hypertrophic cardiomyopathy (HCM)	3.42E-02	LMNA, ITGA3, CACNG2, TPM2, TPM3
Insulin signaling pathway	4.37E-02	MAP2K1, CALML3, GYS1, SH2B2, INSR, PCK1
Melanogenesis	4.55E-02	WNT5B, MAP2K1, CALML3, LEF1, GNAS
Thyroid cancer	7.10E-02	MAP2K1, LEF1, TPM3
Vascular smooth muscle	8.35E-02	MAP2K1, CALML3, ADORA2A, GNAS, PLA2G4E
Adherens junction	9.41E-02	LEF1, CTNNA1, INSR, SRC

(c)

Perinatal Alcohol Exposure (DA)					
**KEGG Term**	**P value**	**Genes**	**KEGG Term**	**P value**	**Genes**
**Upregulated**			**Downregulated**		
Ribosome	9.34E-45	RPL18, RPL17, RPL36A, RPL19, RPL14, RPL13, RPLP2, RPS2, RGD1564062, RPS3, RGD1561736, RPS3A, RPLP1, RPL10, FAU, RPL11, RPL12, RPS27A, RPL36AL, RGD1562415, RPL35A, RGD1563459, LOC681260, RPS4X, RGD1564744, RPS18, RPS19, RPL41, RPS16, RPS17, RPS14, RPS15, RPS12, RPS13, RPS11, UBA52, LOC500371, RPL27A, RPL35, RPL36, RPS15A, RPL37, RPL38, RPL39, RPS25, RPS26, RPL30, RPS27, RPS29, RPL32, RPL7, RPL31, RPL6, RPL34, RPL9, RPL37A-PS1, RPL8, RPL7A, RPS20, RPL4, RPL10A, RPS21, RPS23, RPS24, RGD1560633, RPSA, RPL26, RPS9, RPL24, RPL23A, RPS6, RPS5, RPS8, RPL23, RPL18A, RPL22, RPL13A, RPL21, RPL37A	Calcium signaling pathway	5.86E-03	GNA14, ADORA2B, CYSLTR1, TNNC1, TACR1, PDE1C, HRH2, PDE1A, NOS3, TRPC1, SLC8A1, MYLK3, PHKG2, GRIN1, PRKCG, NTSR1, CACNA1S, P2RX5, P2RX6, AVPR1B, PLCG2, RYR1, ADRA1B, RYR2, ADRA1A, CACNA1C, HTR2A
Huntington's disease	2.01E-10	ATP5D, UQCRC2, ATP5E, CLTA, UQCRC1, AP2S1, CYC1, REST, CLTC, COX5B, UQCRQ, NDUFS7, NDUFS6, NDUFS5, AP2B1, PLCB3, RGD1566212, CREB3L2, DLG4, ATP5H, NDUFA10L1, NDUFB10, NDUFC2, COX4I1, NDUFA10, COX6C, EP300, UQCRH, LOC684509, NDUFB3, NDUFB4, POLR2G, LOC680288, POLR2E, NDUFB7, POLR2L, NDUFB8, NDUFB9, COX7B, ATP5G2, COX7A2L, ATP5G1, POLR2B, COX6B2, AP2M1, NDUFA4, COX7A2, NDUFA8, NDUFA6, NDUFA7, CREBBP, GRIN1, SOD1, VDAC2, NDUFA1, VDAC3, NDUFV3, AP2A2, NDUFV1, BAX, SDHD, COX6A1, ATP5A1	Basal cell carcinoma	9.77E-03	FZD8, DVL3, SMO, BMP2, WNT4, WNT5B, LEF1, WNT9A, GLI2, GLI3, WNT2B, WNT5A, WNT1, BMP2, WNT10B, GSK3B, SUFU, CTNNB1
Alzheimer's disease	3.45E-10	ATP5D, UQCRC2, ATP5E, UQCRC1, CYC1, IDE, COX5B, UQCRQ, NDUFS7, NDUFS6, NDUFS5, APP, PLCB3, LOC303448, RGD1566212, APOE, GRIN2C, MAPT, PSENEN, ATP5H, NDUFA10L1, NDUFB10, NDUFC2, COX4I1, NDUFA10, COX6C, MAPK1, PSEN1, UQCRH, LOC684509, PSEN2, NDUFB3, NDUFB4, LOC680288, NDUFB7, NDUFB8, NDUFB9, COX7B, PPP3R1, ATP5G2, COX7A2L, ATP5G1, TNFRSF1A, PPP3CC, COX6B2, PPP3CA, NDUFA4, COX7A2, NDUFA8, NDUFA6, NDUFA7, GRIN1, BAD, NDUFA1, NCSTN, ATF6, NDUFV3, LRP1, ATP2A2, NDUFV1, GSK3B, SDHD, COX6A1, ATP5A1	Hedgehog signaling pathway	1.12E-02	SMO, BMP2, WNT4, WNT5B, WNT9A, GLI2, LRP2, GLI3, ZIC2, WNT2B, BMP6, WNT5A, WNT1, BMP2, WNT10B, CSNK1G1, GSK3B, SUFU, BMP8A
Parkinson's disease	1.11E-09	UQCRC2, ATP5D, ATP5E, UQCRC1, UCHL1, CYC1, PINK1, COX5B, UQCRQ, NDUFS7, NDUFS6, NDUFS5, RGD1566212, ATP5H, NDUFA10L1, NDUFB10, UBE2J1, NDUFC2, COX4I1, NDUFA10, COX6C, UQCRH, LOC684509, UBC, UBB, NDUFB3, NDUFB4, LOC680288, NDUFB7, NDUFB8, NDUFB9, COX7B, TH, ATP5G2, ATP5G1, COX7A2L, COX6B2, NDUFA4, COX7A2, NDUFA8, NDUFA6, NDUFA7, VDAC2, VDAC3, NDUFA1, PARK7, NDUFV3, UBA1, NDUFV1, SDHD, COX6A1, ATP5A1	Tight junction	1.60E-02	CLDN17, F11R, MAGI2, MAGI1, MPP5, MYH4, MYH6, CTNNB1, CSNK2A2, MYH13, PARD6G, TJP3, JAM2, AKT3, MLLT4

Oxidative phosphorylation	3.19E-09	UQCRC2, ATP5D, ATP5E, UQCRC1, ATP6AP1, CYC1, UQCRQ, COX5B, NDUFS7, NDUFS6, NDUFS5, RGD1566212, ATP5L, ATP5I, COX17, ATP5H, NDUFA10L1, NDUFB10, NDUFC2, COX4I1, NDUFA10, ATP6V1F, COX6C, UQCRH, LOC684509, NDUFB3, NDUFB4, LOC680288, NDUFB7, NDUFB8, NDUFB9, COX7B, ATP5G2, ATP5G1, COX7A2L, COX6B2, NDUFA4, TCIRG1, COX7A2, NDUFA8, NDUFA6, NDUFA7, LHPP, NDUFA1, NDUFV3, NDUFV1, SDHD, COX6A1, ATP5A1	Pancreatic cancer	1.60E-02	ACVR1B, TGFBR2, RAC1, CDK6, MAPK8, BCL2L1, STAT1, FIGF, RALGDS, AKT3
Pancreatic cancer	7.87E-08	MAP2K1, TGFBR1, TP53, SMAD3, BAD, MAPK10, STAT1, TGFB2, VEGFB, CDC42, MAPK1, VEGFC, CCND1, MAPK3, VEGFA, TGFA, PIK3R5, IKBKB, EGF	Notch signaling pathway	2.10E-02	HES1, DVL3, CIR1, APH1A, DTX3L, MAML1, DTX3, RFNG, MAML3, RBPJL
Endocytosis	2.01E-05	CLTA, CHMP5, AP2S1, VPS37A, VPS37B, VPS37C, CLTC, CDC42, SMAP2, AP2B1, ATG2A, GIT2, AGAP2, RT1-T24-4, EGFR, PLD1, PSD2, RT1-EC2, RT1.AA, RT1-A3, RT1-A2, RT1-A1, CHMP1A, ARRB2, ACAP3, PSD, ARRB1, ACAP2, PDGFRA, ARFGAP1, FGFR2, PARD3, FGFR3, SRC, CHMP2B, SH3GLB1, RPL9, RT1-M6-2, HSPA8, CSF1R, AP2M1, STAMBP, GIT1, RT1-CE16, RT1-CE1, RT1-CE13, RT1-CE4, RT1-CE14, KDR, AP2A2, RAB31, RAB22A, HGS, DNM2, RT1-N2, DNM3, EPN3, DNM1L, CLTB, RT1-M3-1, ERBB4, RT1-CE7, TGFBR1, ADRBK2, ADRBK1, RT1-EC2, CDC42, RT1-T18, UBE2M, VPS28, EGF, VPS36, AGAP3, EPN2	Pathways in cancer	2.32E-02	WNT5A, FGFR1, EGLN3, NFKBIA, BCL2L1, SUFU, CTNNB1, CCNE2, WNT1, IGF1R, ACVR1B, RAC1, FAS, FGF2, FIGF, AKT3, BMP2, WNT10B, HSP90AA1, RXRA, TGFBR2, FGF23, CDK6, STAT1, RALGDS, DAPK1, GSK3B, MAPK8
Neurotrophin signaling pathway	1.81E-04	IRAK2, YWHAZ, MAP2K1, CAMK2G, TP53, BAD, MAPK10, CDC42, MAPK1, YWHAH, CALML3, MAPK14, MAP3K1, NTRK2, MAPK3, RHOA, PIK3R5, RHOC, IKBKB, MAP2K7, MAP2K5, YWHAZ, GRB2, CAMK2G, FOXO3, MAPKAPK2, CDC42, SOS2, PIK3CA, SHC1, NGFRAP1, CSK, MAP2K7, PIK3R1, ARHGDIA, ARHGDIB, AKT2, YWHAB, BAD, YWHAE, RPS6KA5, NTRK3, MAPK1, YWHAG, RPS6KA1, PSEN1, BAX, GSK3B, MAPK14, NTRK2, YWHAQ, MAPK9, RAP1B	Fc epsilon RI signaling pathway	2.62E-02	AKT1, IL4, IL5, KRAS, GAB2, FYN, PLCG2, PLA2G2A, IL13, PLA2G2C, PRKCG, VAV2, AKT2
Bladder cancer	2.18E-04	VEGFB, MAPK1, VEGFC, CCND1, TYMP, MAP2K1, MAPK3, VEGFA, TP53, EGF	Vascular smooth muscle contraction	2.94E-02	RAMP3, ACTC1, ADORA2B, ROCK1, MYLK3, NPR1, PRKCG, CACNA1S, ARHGEF11, ACTG2, CYP4X1, AVPR1B, PLA2G2A, ADRA1B, ADRA1A, PLA2G2C, CACNA1C
Renal cell carcinoma	3.12E-04	EPAS1, GRB2, PDGFA, VHL, CREBBP, FLCN, TGFB2, RBX1, CDC42, CUL2, MAPK1, HIF1A, EP300, ETS1, SLC2A1, SOS2, VEGFA, TCEB2, PIK3CA, RAP1B, PIK3R1, AKT2, VEGFB, CDC42, MAPK1, VEGFC, EPAS1, MAP2K1, MAPK3, VEGFA, TGFA, PIK3R5, TGFB2, ARNT	Neuroactive ligand-receptor interaction	3.69E-02	F2RL2, CGA, TAAR7E, ADORA2B, CYSLTR1, TACR1, F2RL1, LPAR2, GCGR, S1PR2, KISS1R, HRH2, ADRA2A, GRID1, PTGER4, GRIN1, BRS3, GRIA4, NTSR1, FSHR, LEP, P2RX5, GPR35, P2RX6, AVPR1B, ADRA1B, ADRA1A, FSHB, GHSR, GLP1R, FPR2L, HTR2A
Antigen processing and presentation	7.29E-04	HSP90AB1, PDIA3, IFI30, RT1-DMB, CALR, CANX, B2M, LOC499644, LOC679973, HSPA4, HSPA5, RT1-M6-2, RT1-T24-4, HSPA8, RT1-CE16, HSP90AA1, RT1-CE1, RFX5, RT1-CE4, RT1-CE13, RT1-CE14, CTSS, RT1-EC2, RT1.AA, RT1-A3, RT1-A2, RT1-A1, PSME2, CTSB, RT1-N2	Axon guidance	4.07E-02	ROCK1, GNAI1, NTNG1, SLIT1, CXCL12, RND1, KRAS, SEMA4G, CXCR4, FYN, ROBO1, SEMA3F, SRGAP3, SEMA3C, EFNA5, SEMA3A, ROBO3, PAK1
Proteasome	8.68E-04	PSMB10, SHFM1, PSMA7, PSMB4, PSMC6, PSMB6, PSMC5, PSMA6, PSMD11, PSME2, PSMA4, PSMC2, PSMD1, PSMD4, PSME4, PSMD6, PSMD7	Asthma	4.11E-02	IL4, IL5, EPX, IL13, RT1-DOA, IL10

GnRH signaling pathway	1.44E-03	ADCY2, MAP2K1, ADCY5, CAMK2G, MAPK10, CDC42, MAPK1, CALML3, MAPK14, MAP3K1, MAPK3, PLA2G2C, PRKACB, PLA2G3, MAP2K7	Olfactory transduction	4.48E-02	OLR1156, OLR1151, OLR1091, OLR1349, OLR23, OLR463, OLR466, OLR263, OLR655, OLR1200, OLR1096, CLCA2, OLR1165, OLR1601, OLR35, OLR1605, OLR702, OLR705, OLR251, OLR646, OLR1684, OLR103, OLR592, OLR1742, OLR105, OLR1749, OLR578, OLR1365, OLR1468, OLR479, LOC682961, OLR44, OLR1557, OLR486, OLR485, OLR285, OLR302, OLR282, OLR1458, OLR881, OLR321, OLR1455, OLR1454, OLR1147, OLR767, OLR1563, OLR1767, OLR1306, OLR889, OLR1307, OLR234, OLR62, OLR378, OLR329, LOC687881, OLR1102, OLR1249, OLR1393, OLR327, OLR1394, OLR1252, OLR1106, OLR1643, OLR404, OLR604, OLR1504, OLR550, OLR1701, OLR1585, OLR410, OLR192, LOC686288, OLR1265, OLR1329, OLR1514, OLR1425, OLR1179, OLR1228, OLR1509, PDE1C, OLR1699, OLR1071, OLR440, OLR1607, OLR206, OLR1609, OLR425, OLR429, OLR721, OLR1186, OLR1523, OLR1522, OLR360, OLR527, OLR1413, OLR1388, OLR1387, OLR1409, OLR1283
Colorectal cancer	3.23E-03	DCC, MAP2K1, TGFBR1, TP53, SMAD3, BAD, MAPK10, TGFB2, MAPK1, CCND1, MAPK3, PIK3R5, AXIN1, EGFR, APC2, GRB2, BAD, FZD7, TGFB2, DVL1, MAPK1, GSK3B, BAX, SOS2, PDGFRA, PIK3CA, MAPK9, PDGFRB, PIK3R1, AKT2	Regulation of actin cytoskeleton	5.26E-02	LOC316717, GNA13, FGFR1, VAV3, PIP5K1B, ITGA11, FGF23, NCKAP1L, INSRR, PAK2, PAK3, PAK4, RAC1, PPP1R12A, ITGAD, PIP4K2A, FGF2, FGD3, F2R
Fatty acid metabolism	3.61E-03	GCDH, ACADSB, ACADS, ADH5, ACADL, ACAT1, HADHA, ACOX3, ACSL1, ALDH2, HADH, ACSL3, ALDH9A1, ACSL6	ErbB signaling pathway	5.28E-02	PAK2, PAK3, PAK4, GSK3B, BTC, CAMK2D, MAPK8, CAMK2A, ABL2, AKT3
Long-term potentiation	3.88E-03	CAMK2G, GRIN1, CREBBP, PPP3R1, PPP1CC, PRKX, PRKCB, MAPK1, PLCB3, PPP1CA, EP300, RPS6KA1, GRIA1, GRIN2C, PPP1R1A, PPP3CC, PRKACA, RAP1B, PPP3CA	Adipocytokine signaling pathway	5.61E-02	LEP, AKT1, CD36, RXRB, RELA, JAK2, ACACB, PRKAA2, AKT2, TRADD, PCK1, CPT1C, PPARA, STK11, RXRA, NFKBIB, NFKBIA, MAPK8, AKT3
Adherens junction	4.61E-03	PTPRB, BAIAP2, TGFBR1, SMAD3, CTNNA1, MAP3K7, MAPK1, CDC42, SORBS1, PVRL3, MAPK3, RHOA, RHOC, ACTB, EGFR, PTPN6, FGFR1, PARD3, PTPRF, ACTN4, BAIAP2, NLK, CREBBP, WASF2, SRC, ACTG1, CDC42, MAPK1, EP300, PTPN1, SSX2IP	Cardiac muscle contraction	6.56E-02	LOC688869, FXYD2, ACTC1, SLC8A1, TNNC1, CACNG5, RYR2, CACNA1C, TPM2, CACNA1S, LOC687508, CACNA2D2
VEGF signaling pathway	4.61E-03	CDC42, MAPK1, MAP2K1, MAPK14, MAPK3, VEGFA, MAPKAPK3, PLA2G2C, PIK3R5, BAD, NFATC2, PLA2G3, PPP3R1, MAPKAPK2, BAD, SRC, PXN, KDR, PRKCB, CDC42, MAPK1, MAPK14, VEGFA, PPP3CC, PIK3CA, PPP3CA, PIK3R1, AKT2	Wnt signaling pathway	7.07E-02	WNT5A, WNT10B, NKD2, CTNNB1, CSNK2A2, PEG12, WNT1, CHD8, RGD1564855, GSK3B, RAC1, CAMK2D, MAPK8, CAMK2A
ErbB signaling pathway	4.84E-03	ERBB4, MAP2K1, CAMK2G, BAD, MAPK10, MAPK1, MAPK3, TGFA, PIK3R5, NRG1, EGF, ABL2, MAP2K7	Cytokine-cytokine receptor interaction	7.42E-02	IL4, IL1R2, IL5, IL22RA1, CXCL9, FASLG, IL13, CD70, IL21, CCL4, CXCL12, IL10, IL12RB2, LEP, CCR6, CXCR4, IFNA4, IFNK, NGFR, MPL, CD27, LTA, ACVR1, EPO
Chronic myeloid leukemia	5.12E-03	MAPK1, CCND1, GAB2, MAP2K1, TGFBR1, MAPK3, TP53, SMAD3, PIK3R5, BAD, IKBKB, TGFB2	Drug metabolism	7.67E-02	XDH, UMPS, CYP3A73, NAT1, UGT2A1, CDA, UPP2, UCK2

Chemokine signaling pathway	7.22E-03	ADCY2, MAP2K1, NCF1, ADCY5, BCAR1, ADRBK2, GNG11, ADRBK1, GNG12, CX3CL1, STAT1, CDC42, MAPK1, GNB1, MAPK3, RHOA, PIK3R5, RHOC, PRKACB, IKBKB, GNG7, PARD3, GNAI2, GRB2, FOXO3, PRKX, PXN, CCL27, GNG8, CDC42, PLCB3, SOS2, PIK3CA, PRKACA, SHC1, CSK, PIK3R1, AKT2, ROCK1, LYN, STAT2, PRKCB, CCR9, MAPK1, ARRB2, CXCL14, GNB2, ARRB1, GNG10, GSK3B, CX3CR1, RAP1B, JAK3	Colorectal cancer	9.06E-02	ACVR1B, IGF1R, GSK3B, TGFBR2, RAC1, MAPK8, RALGDS, AKT3, CTNNB1
Thyroid cancer	8.17E-03	MAPK1, CCND1, MAP2K1, MAPK3, TP53, TFG, TPM3	Renal cell carcinoma	9.80E-02	PAK2, PAK3, PAK4, RAC1, EGLN3, FLCN, FIGF, AKT3
Regulation of actin cytoskeleton	8.33E-03	FGFR2, FGFR1, ENAH, FGFR3, APC2, PDGFA, MRAS, SSH3, GNA12, SSH2, WASF2, ABI2, ARPC5, PXN, ACTG1, CDC42, PFN1, EZR, ARPC3, GSN, ITGAV, SOS2, PIK3CA, FGF1, CSK, PIK3R1, ACTB, EGFR, GIT1, ARHGEF1, ROCK1, ACTN4, BAIAP2, ARHGEF12, MYH9, PPP1CC, ARPC1B, MAPK1, PPP1CA, ITGA6, PDGFRA, PDGFRB, TMSB4X			
Pathways in cancer	9.12E-03	DCC, TFG, ITGB1, TGFB2, ARNT, TPM3, CDC42, RHOA, TGFA, PIK3R5, RHOC, EGF, AXIN1, BMP4, EPAS1, MAP2K1, TGFBR1, TP53, SMAD3, MAPK10, BAD, CTNNA1, STAT1, CDK2, VEGFB, VEGFC, MAPK1, WNT7B, CCND1, VEGFA, MAPK3, IKBKB, HSP90AB1, PDGFA, MITF, FOXO1, TGFB2, CDC42, CUL2, LOC499644, SLC2A1, PIK3CA, RALA, TPR, FGF1, AKT2, EGFR, CTBP1, HSP90AA1, CTBP2, RALBP1, PRKCB, JUP, MAPK1, EP300, HIF1A, PIAS4, VEGFA, PDGFRA, PDGFRB, MAPK9, PIAS2, FGFR2, FGFR1, FGFR3, APC2, GRB2, SUFU, TPM3, RBX1, ITGAV, SOS2, LOC679973, PIK3R1, TRAF4, CSF1R, EPAS1, VHL, CREBBP, BAD, FZD7, DVL1, ITGA6, BAX, GSK3B, TCEB2, PTCH1, WNT7A			
Cardiac muscle contraction	9.55E-03	UQCRC2, CACNA2D1, COX7A2, UQCRC1, ATP1B2, COX7B, CYC1, ATP1A3, COX4I1, COX7A2L, ATP1A2, CACNA2D2, COX5B, UQCRQ, COX6C, TPM3, ATP2A2, UQCRH, COX6A1, COX6B2			
MAPK signaling pathway	9.95E-03	MAPKAPK3, GNG12, TGFB2, MAP3K7, CDC42, MAP3K1, JUND, PRKACB, NFATC2, EGF, MAP2K7, MAP2K5, MAP2K1, TAOK1, TGFBR1, TP53, MAPK10, MAPK1, MAPK14, MAPK3, NTRK2, MAPK8IP3, PLA2G2C, STMN1, IKBKB, PLA2G3, DUSP8, PDGFA, GNA12, PRKX, TGFB2, CDC42, MAPT, PRKACA, FGF1, MAP2K7, AKT2, EGFR, ECSIT, DDIT3, PRKCB, MAP4K3, MAP4K4, MAPK1, ARRB2, RASGRF1, ARRB1, PDGFRA, PDGFRB, MAPK9, FGFR2, FGFR1, FGFR3, GRB2, MRAS, PPP3R1, MAPKAPK2, SRF, TNFRSF1A, SOS2, PPP3CC, PPP3CA, HSPA8, CACNA2D1, TAOK2, NLK, TAOK3, CACNA2D2, RPS6KA5, DUSP3, RPS6KA1, MAPK14, NTRK2, MAPK8IP2, MAPK8IP3, RAP1B, MAPK8IP1, DUSP6			
Glioma	1.05E-02	MAPK1, CCND1, MAP2K1, CALML3, CAMK2G, MAPK3, TP53, TGFA, PIK3R5, EGF			

Prostate cancer	1.10E-02	FGFR2, HSP90AB1, EGFR, FGFR1, HSP90AA1, GRB2, PDGFA, CREBBP, FOXO1, BAD, MAPK1, PDPK1, LOC499644, EP300, GSK3B, SOS2, CREB3L2, PDGFRA, PDGFRB, PIK3CA, LOC679973, PIK3R1, AKT2, MAPK1, CCND1, MAP2K1, CREB1, MAPK3, TP53, TGFA, PIK3R5, BAD, EGF, IKBKB, CDK2	
Non-small cell lung cancer	1.22E-02	MAPK1, CCND1, MAP2K1, MAPK3, TP53, TGFA, PIK3R5, BAD, EGF	
Galactose metabolism	1.30E-02	GALK2, B4GALT2, PGM1, GAA, PFKM, LCT	
Focal adhesion	1.39E-02	MAP2K1, BCAR1, ITGA1, MYLK2, BAD, MAPK10, ITGB1, COL5A2, COL5A1, VEGFB, CDC42, VEGFC, MAPK1, CCND1, MAPK3, ILK, VEGFA, RHOA, PIK3R5, RHOC, EGF, MYLK	
Spliceosome	1.42E-02	DHX8, CWC15, SNRPC-PS1, PRPF3, PRPF18, PRPF4, HNRNPU, EIF4A3, AQR, HNRNPK, DHX38, USP39, PRPF40A, DDX42, SNRPG	
Gap junction	1.43E-02	EGFR, GNAI2, TUBB2B, GRB2, PDGFA, TUBB2A, TUBA3B, GJA1, PRKX, SRC, PRKCB, MAPK1, PLCB3, CSNK1D, SOS2, TUBB5, PDGFRA, PDGFRB, PRKACA, GNAS, TUBA1A, TUBA1B	
Notch signaling pathway	1.47E-02	DTX4, CTBP1, CTBP2, CREBBP, JAG2, DVL1, NCSTN, EP300, PSEN1, HES5, PSEN2, PSENEN, LFNG, NCOR2, APH1A, DTX2, PSEN2, NOTCH4, NUMB, RBPJ, NUMBL	
Fc gamma R-mediated phagocytosis	1.66E-02	CDC42, MAPK1, DNM3, MYO10, DNM1L, GAB2, MAP2K1, LIMK1, NCF1, ARPC5L, MAPK3, PIK3R5, PLD1, LYN, MARCKSL1, WASF2, ARPC5, VASP, PRKCB, CDC42, ARPC1B, MAPK1, ARPC3, GSN, FCGR1A, PIK3CA, FCGR2A, PPAP2B, PIK3R1, DNM2, AKT2	
Insulin signaling pathway	2.47E-02	GRB2, FOXO1, PRKX, PDPK1, SOS2, FASN, PIK3CA, PRKACA, SHC1, PIK3R1, AKT2, PTPRF, FLOT1, ACACA, PRKAB1, BAD, RPS6, PCK2, PPP1CC, MAPK1, PPP1CA, TSC1, GSK3B, PRKAR1A, TSC2, MAPK9, RHEB, PTPN1	
Axon guidance	2.69E-02	ABLIM2, GNAI2, PPP3R1, L1CAM, LRRC4C, CDC42, ROBO1, PPP3CC, PPP3CA, ROCK1, PLXNB1, EFNB2, DPYSL2, ARHGEF12, NTN1, SLIT1, MAPK1, SEMA6B, EPHA6, SEMA6D, RGS3, GSK3B, SEMA4B, EFNA4, SEMA4D, SEMA4A, SRGAP2	
mTOR signaling pathway	2.77E-02	STRADA, RPS6, MAPK1, PDPK1, HIF1A, RPS6KA1, TSC1, TSC2, VEGFA, RHEB, PIK3CA, MLST8, PIK3R1, AKT2, VEGFB, EIF4B, MAPK1, VEGFC, ULK1, MAPK3, VEGFA, PIK3R5	
Lysosome	3.19E-02	CLTA, AP4E1, ATP6AP1, ABCA2, CTSA, ACP2, CLTC, SLC11A2, LAPTM5, GNPTAB, MAN2B1, GBA, TCIRG1, PSAP, CTSS, CD63, FUCA1, M6PR, LAMP1, SLC17A5, LAMP2, IGF2R, CTSD, CTSB, GGA1, NAGPA, CLTB, LIPA, ARSG, GUSB, SMPD1, CTSE, GAA, PPT1, SCARB2, CD164, CTSH, ATP6V0D2	
Glycerophospholipid metabolism	3.24E-02	CRLS1, DGKB, DGKG, CHKB, PLA2G2C, ETNK2, PLA2G3, AGPAT3, CHPT1	
TGF-beta signaling pathway	3.42E-02	BMP4, MAPK1, PPP2R1A, ACVR2B, ID2, TGFBR1, MAPK3, RHOA, BMPR2, SMAD3, RHOC, TGFB2	

Fc epsilon RI signaling pathway	3.92E-02	MAPK1, GAB2, MAP2K1, MAPK14, MAPK3, PLA2G2C, PIK3R5, MAPK10, PLA2G3, MAP2K7	
Vascular smooth muscle contraction	4.00E-02	ADCY2, MAP2K1, ADCY5, MYLK2, MAPK1, CALML3, MAPK3, RHOA, PLA2G2C, RHOC, PRKACB, PLA2G3, ADRA1D, MYLK	
Dorso-ventral axis formation	4.08E-02	MAPK1, MAP2K1, MAPK3, NOTCH4, SPIRE1	
Ubiquitin mediated proteolysis	4.17E-02	ANAPC5, VHL, ANAPC4, UBE2J1, CDC23, SAE1, UBE2I, ANAPC11, UBOX5, SKP1, CDC34, CDC26, UBE2B, STUB1, RBX1, RFWD2, CUL2, UBE2D3, CUL5, PIAS4, MGRN1, UBE2K, UBA1, TCEB2, PIAS2, LOC689226	
Toll-like receptor signaling pathway	4.47E-02	MAP3K7, MAPK1, MAP2K1, MAPK14, IRF7, MAPK3, PIK3R5, MAPK10, IKBKB, STAT1, MAP2K7	
Lysine degradation	5.18E-02	SUV420H2, GCDH, EHMT1, PLOD2, ALDH2, SETD8, HADH, NSD1, ACAT1, HADHA, ALDH9A1	
Ether lipid metabolism	5.20E-02	ENPP2, PLA2G7, PLA2G2C, PLA2G3, AGPAT3, CHPT1	
DNA replication	5.78E-02	RPA1, MCM7, LIG1, POLD1, MCM4, FEN1	
Wnt signaling pathway	6.97E-02	APC2, CAMK2G, PPP2R5C, PPP3R1, DAAM1, DAAM2, PRKX, RBX1, PLCB3, PPP3CC, PRKACA, PPP3CA, CSNK1A1, CTBP1, CTBP2, ROCK1, NLK, CREBBP, SKP1, FZD7, PORCN, DVL1, PRKCB, EP300, PSEN1, GSK3B, MAPK9, WNT7A	
Steroid biosynthesis	7.73E-02	CYP51, TM7SF2, SOAT1, SQLE, DHCR7, NSDHL	
N-Glycan biosynthesis	7.83E-02	MAN2A2, B4GALT3, GANAB, STT3A, RPN1, MAN1B1, DPM2, DPM3, MAN1A1, RPN2, ALG11	
NOD-like receptor signaling pathway	7.96E-02	MAP3K7, MAPK1, MAPK14, MAPK3, PSTPIP1, PYCARD, MAPK10, IKBKB	
Propanoate metabolism	8.00E-02	LDHB, ACSS1, MLYCD, SUCLG1, ALDH2, ACACA, ACAT1, HADHA, ALDH9A1	

(d)

Perinatal Alcohol Exposure (non-DA)		
**KEGG Term**	**P value**	**Genes**
**Upregulated**		
Aldosterone-regulated sodium reabsorption	1.43E-03	ATP1B2, MAPK3, IGF1, PIK3R5, ATP1A2, PIK3R1
Regulation of actin cytoskeleton	7.73E-03	ACTB, GNA13, FGFR3, PDGFA, GNA12, MAPK3, WASF2, CYFIP1, PIK3R5, PIK3R1, FGF4
Melanoma	1.11E-02	PDGFA, MAPK3, IGF1, PIK3R5, PIK3R1, FGF4
Natural killer cell mediated cytotoxicity	1.35E-02	MAPK3, PPP3R1, FCER1G, PIK3R5, IFNGR1, PIK3R1, TYROBP
Cardiac muscle contraction	1.82E-02	UQCRC2, ATP2A2, ATP1B2, COX7C, ATP1A2, TPM3
Antigen processing and presentation	2.90E-02	RT1-CE1, PDIA3, CD4, CTSS, B2M, RT1-N2
Glioma	3.14E-02	PDGFA, MAPK3, IGF1, PIK3R5, PIK3R1
Renal cell carcinoma	4.62E-02	PDGFA, MAPK3, GAB1, PIK3R5, PIK3R1
Pancreatic cancer	4.62E-02	PLD1, MAPK3, JAK1, PIK3R5, PIK3R1
Pathways in cancer	4.63E-02	FGFR3, PDGFA, MAPK3, IGF1, JAK1, PIK3R5, PTCH1, PIK3R1, FGF4, CSF1R, TPM3, AXIN1
Jak-STAT signaling pathway	5.54E-02	CSF3, OSMR, LIFR, JAK1, PIK3R5, IFNGR1, PIK3R1
T cell receptor signaling pathway	6.29E-02	MAPK3, PPP3R1, PIK3R5, CD4, PIK3R1, CD28
mTOR signaling pathway	8.56E-02	MAPK3, IGF1, PIK3R5, PIK3R1
Fc gamma R-mediated phagocytosis	9.45E-02	PLD1, MAPK3, WASF2, PIK3R5, PIK3R1
**Downregulated**		
Vascular smooth muscle contraction	1.69E-02	CYP4A2, MAP2K2, CALML3, ADORA2A, CALM3, GNAS, ARHGEF12, PLA2G4E, PRKCB
RNA degradation	2.45E-02	EXOSC9, CNOT3, EDC4, ENO3, LSM3, LSM2
Pathways in cancer	4.24E-02	AR, COL4A1, WNT5B, RXRB, PDGFA, MAP2K2, CYCT, LEF1, ITGA3, FGF20, CTNNA1, PRKCB, VEGFC, LOC499644, PIAS4, RASSF1
Long-term potentiation	4.49E-02	RPS6KA6, MAP2K2, CALML3, PPP1R1A, CALM3, PRKCB
Melanogenesis	5.11E-02	WNT5B, MAP2K2, CALML3, LEF1, CALM3, GNAS, PRKCB
Arrhythmogenic right ventricular cardiomyopathy (ARVC)	6.39E-02	PKP2, LMNA, LEF1, ITGA3, CACNG2, CTNNA1
Insulin signaling pathway	9.00E-02	GCK, MAP2K2, CALML3, CALM3, RPS6KB2, INS1, INSR, PCK1

Nicotine-Alcohol (DA) Upregulated

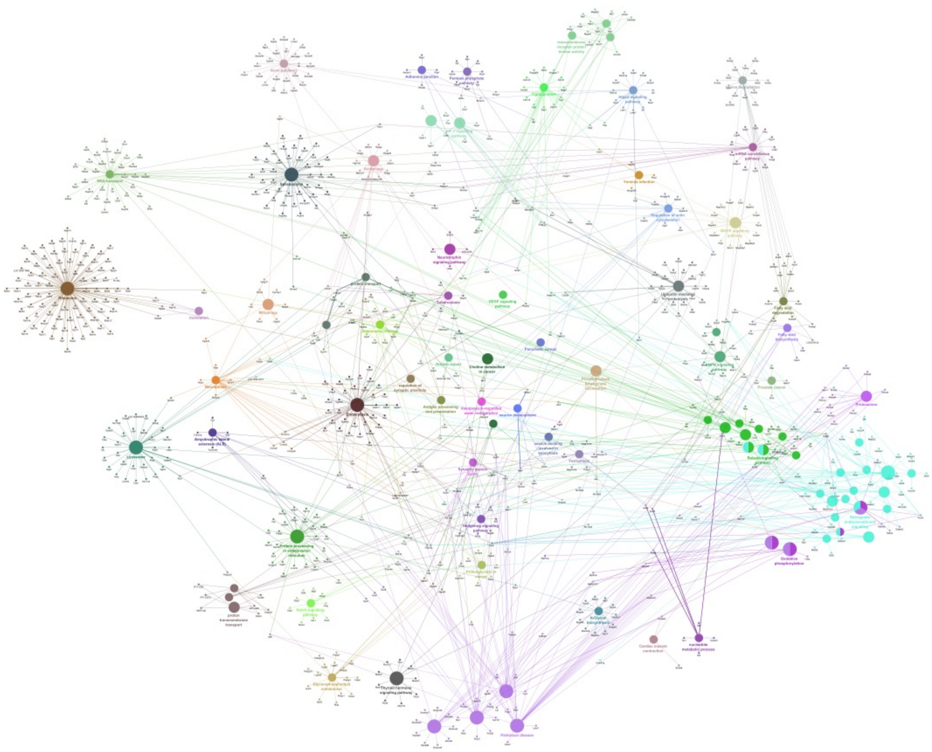

**Nicotine-Alcohol DA**	**adjusted p value**
Upregulated	
Ribosome	1.68E-21
Parkinson disease	3.33E-17
Huntington disease	2.50E-10
Thermogenesis	1.75E-08
Oxidative phosphorylation	2.53E-08
Alzheimer disease	3.35E-08
Non-alcoholic fatty liver disease (NAFLD)	5.02E-07
Protein processing in endoplasmic reticulum	4.45E-06
Endocytosis	1.20E-05
Retrograde endocannabinoid signaling	2.71E-05
Glutamatergic synapse	4.91E-05
Lysosome	0.0001
Thyroid hormone signaling pathway	0.0001
Spliceosome	0.0002
AMPK signaling pathway	0.0014
Proximal tubule bicarbonate reclamation	0.0014
Mitophagy	0.0016
HIF-1 signaling pathway	0.0016
Relaxin signaling pathway	0.0025
Autophagy	0.0027
Hepatitis B	0.0028
Ubiquitin mediated proteolysis	0.0031
Human cytomegalovirus infection	0.0031
Renal cell carcinoma	0.0031
Neurotrophin signaling pathway	0.0032
Choline metabolism in cancer	0.0034
Endocrine and other factor-regulated calcium reabsorption	0.0034
Viral carcinogenesis	0.0035
Long-term potentiation	0.0036
MAPK signaling pathway	0.0041
Proteasome	0.0042
Proton transmembrane transport	0.0049
GABAergic synapse	0.0049
RNA transport	0.0052
Dopaminergic synapse	0.0054
Protein export	0.0056
Amyotrophic lateral sclerosis (ALS)	0.0057
Gastric acid secretion	0.0061
Human T-cell leukemia virus 1 infection	0.0061
Yersinia infection	0.0073
Hippo signaling pathway	0.0086
Hedgehog signaling pathway	0.0088
Cardiac muscle contraction	0.0088
Adherens junction	0.0098
Regulation of exocytosis	0.0105
Osteoclast differentiation	0.0114
Insulin secretion	0.0114
Amphetamine addiction	0.0117

**Nicotine-Alcohol DA**	**adjusted p value**
Upregulated	
Alcoholism	0.0117
Protein transport	0.0120
Lysine degradation	0.0121
Ferroptosis	0.0133
Glycerophospholipid metabolism	0.0133
Salmonella infection	0.0133
Necroptosis	0.0133
Axon guidance	0.0139
Synaptic vesicle cycle	0.0141
Phagosome	0.0144
Tight junction	0.0159
Kaposi sarcoma-associated herpesvirus infection	0.0160
Vasopressin-regulated water reabsorption	0.0167
Cellular senescence	0.0197
VEGF signaling pathway	0.0205
Fatty acid biosynthesis	0.0206
Proton transmembrane transporter activity	0.0207
Translation	0.0211
Intracellular protein transport	0.0211
Fatty acid degradation	0.0219
Cation transmembrane transporter activity	0.0224
Pentose phosphate pathway	0.0233
Proteoglycans in cancer	0.0264
Regulation of synaptic plasticity	0.0274
Neuron development	0.0294
Nucleotide metabolic process	0.0294
Transmembrane receptor protein kinase activity	0.0294
Vesicle docking involved in exocytosis	0.0309
Regulation of actin cytoskeleton	0.0311
Protein serine/threonine kinase activity	0.0337
Pancreatic cancer	0.0341
mRNA surveillance pathway	0.0367
Protein kinase activity	0.0372
Phospholipase D signaling pathway	0.0372
Prostate cancer	0.0386
Notch signaling pathway	0.0394
Circadian entrainment	0.0394
Longevity regulating pathway	0.0396
cGMP-PKG signaling pathway	0.0404
Tuberculosis	0.0405
Antigen processing and presentation	0.0409
Adrenergic signaling in cardiomyocytes	0.0418
N-Glycan biosynthesis	0.0458
Oxytocin signaling pathway	0.0463
Leishmaniasis	0.0467
Salivary secretion	0.0496

Supplementary Figure S1. Functional enrichment analysis of upregulated DEGs. DEG lists following perinatal nicotine-alcohol on DA neurons. GO biological processes and KEGG pathway analysis results are listed in the accompanied tables with BH-corrected p-values.

Nicotine-Alcohol (non-DA) Upregulated

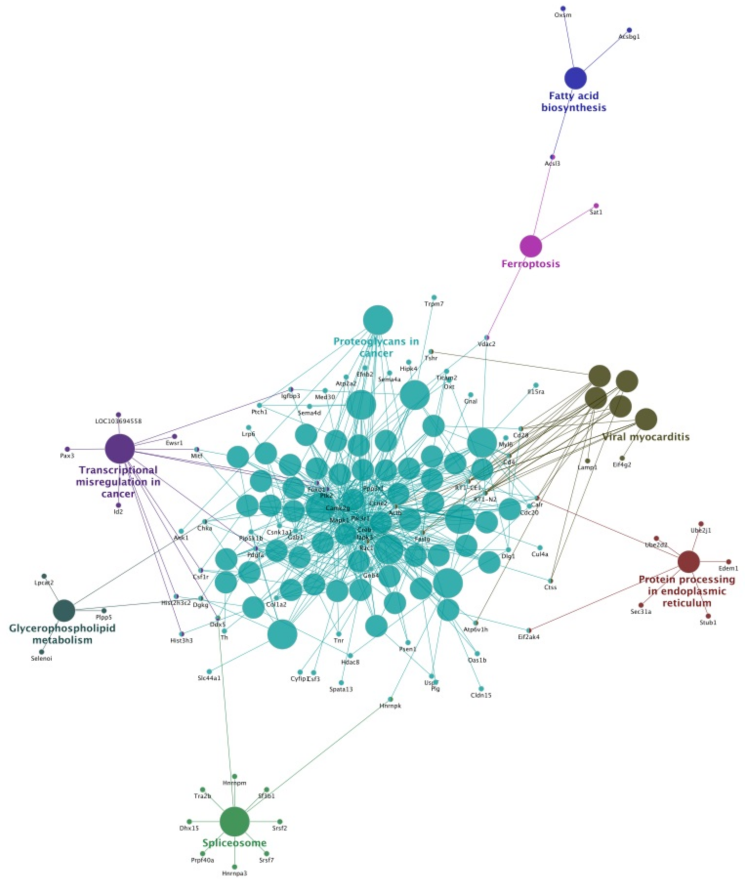

Nicotine-Alcohol NDA *Upregulated*	adjusted p value
Proteoglycans in cancer	0.0022
Choline metabolism in cancer	0.0023
Transcriptional misregulation in cancer	0.0024
Spliceosome	0.0025
Viral carcinogenesis	0.0026
Cellular senescence	0.0026
Human T-cell leukemia virus 1 infection	0.0026
Axon guidance	0.0036
Human immunodeficiency virus 1 infection	0.0037
VEGF signaling pathway	0.0045
Human papillomavirus infection	0.0046
Phosphorylation	0.0075
PD-L1 expression and PD-1 checkpoint pathway in cancer	0.0078
Human cytomegalovirus infection	0.0096
Prostate cancer	0.0096
Neurotrophin signaling pathway	0.0099
Regulation of cellular localization	0.0109
T cell receptor signaling pathway	0.0133
Focal adhesion	0.0138
Kinase activity	0.0140
Fatty acid biosynthesis	0.0153
Regulation of localization	0.0157
ErbB signaling pathway	0.0164
cAMP signaling pathway	0.0169
Thyroid hormone signaling pathway	0.0174
Regulation of actin cytoskeleton	0.0175
PI3K-Akt signaling pathway	0.0177
Viral myocarditis	0.0197
Yersinia infection	0.0203
Osteoclast differentiation	0.0224
Renal cell carcinoma	0.0225
Natural killer cell mediated cytotoxicity	0.0225
Melanoma	0.0231
Regulation of transport	0.0243
Bacterial invasion of epithelial cells	0.0244
Glioma	0.0244
Phosphate-containing compound metabolic process	0.0244
AGE-RAGE signaling pathway in diabetic complications	0.0247
Chagas disease (American trypanosomiasis)	0.0268
Phosphorus metabolic process	0.0302
Alcoholism	0.0303
Cholinergic synapse	0.0305
Protein phosphorylation	0.0306
Autoimmune thyroid disease	0.0308
B cell receptor signaling pathway	0.0308
Growth hormone synthesis, secretion and action	0.0326
Gastric cancer	0.0328
Phospholipase D signaling pathway	0.0332

Nicotine-Alcohol NDA *Upregulated*	adjusted p value
Intracellular signal transduction	0.0333
Hepatitis C	0.0337
Oxytocin signaling pathway	0.0340
Phagosome	0.0370
Hepatitis B	0.0374
Protein processing in endoplasmic reticulum	0.0423
Toll-like receptor signaling pathway	0.0425
Fc gamma R-mediated phagocytosis	0.0425
Relaxin signaling pathway	0.0434
Influenza A	0.0440
Long-term potentiation	0.0440
Amphetamine addiction	0.0446
Acute myeloid leukemia	0.0447
FoxO signaling pathway	0.0447
Glycerophospholipid metabolism	0.0450
Aldosterone-regulated sodium reabsorption	0.0452
Antigen processing and presentation	0.0452
Fc epsilon RI signaling pathway	0.0453
Graft-versus-host disease	0.0474
Adherens junction	0.0474
Apoptosis	0.0474
Ferroptosis	0.0475

Supplementary Figure S2. Functional enrichment analysis of upregulated DEGs. DEG lists following perinatal nicotine-alcohol on non-DA neurons. GO biological processes and KEGG pathway analysis results are listed in the accompanied tables with BH-corrected p-values.

Alcohol (DA) Upregulated

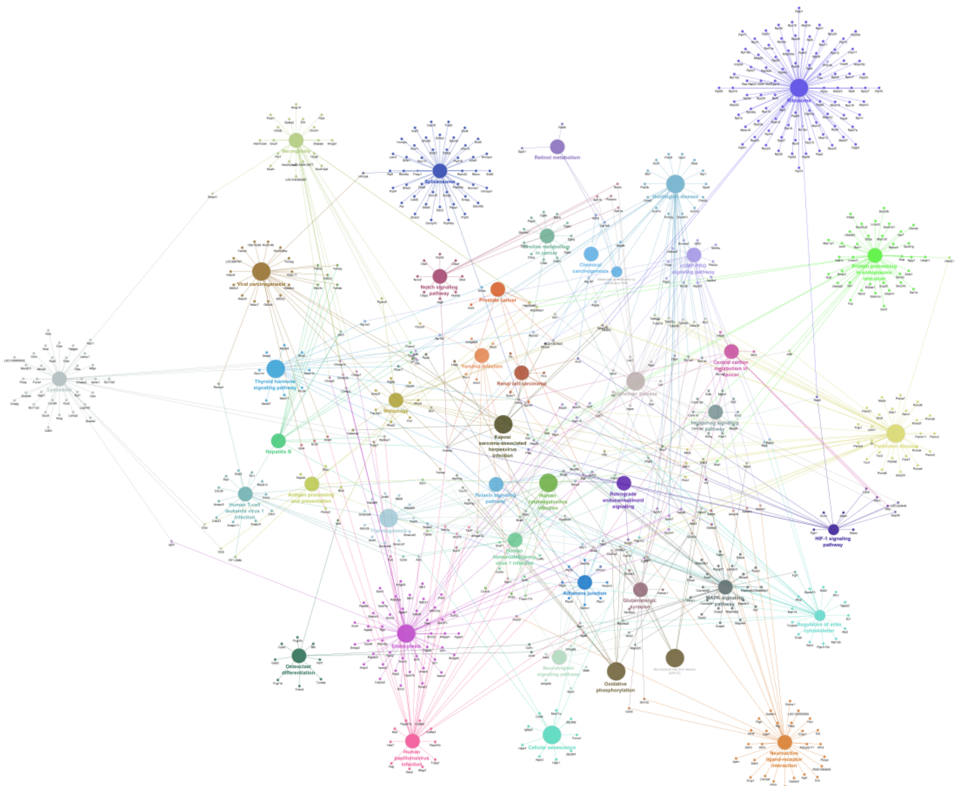

**Alcohol DA**	**adjusted p value**
Upregulated	
Ribosome	5.97E-21
Parkinson disease	4.80E-14
Huntington disease	2.29E-09
Endocytosis	5.65E-09
Alzheimer disease	6.37E-07
Oxidative phosphorylation	1.51E-06
Thermogenesis	1.63E-06
Viral carcinogenesis	6.82E-06
Thyroid hormone signaling pathway	9.74E-06
Kaposi sarcoma-associated herpesvirus infection	1.46E-05
Human cytomegalovirus infection	6.33E-05
Cellular senescence	1.67E-04
Non-alcoholic fatty liver disease (NAFLD)	2.79E-04
Human T-cell leukemia virus 1 infection	5.26E-04
Retrograde endocannabinoid signaling	5.35E-04
Adherens junction	7.47E-04
Renal cell carcinoma	7.47E-04
Mitophagy	9.09E-04
Spliceosome	0.0010
Prostate cancer	0.0011
Hedgehog signaling pathway	0.0013
Osteoclast differentiation	0.0020
Protein processing in endoplasmic reticulum	0.0021
MAPK signaling pathway	0.0023
Hepatitis B	0.0023
Choline metabolism in cancer	0.0023
Chemical carcinogenesis	0.0024
Lysosome	0.0024
Human papillomavirus infection	0.0025
Glutamatergic synapse	0.0025
Antigen processing and presentation	0.0026
Neurotrophin signaling pathway	0.0027
Yersinia infection	0.0027
Human immunodeficiency virus 1 infection	0.0027
Notch signaling pathway	0.0035
Relaxin signaling pathway	0.0036
Neuroactive ligand-receptor interaction	0.0036
cGMP-PKG signaling pathway	0.0040
Necroptosis	0.0044
Central carbon metabolism in cancer	0.0049
HIF-1 signaling pathway	0.0052
Regulation of actin cytoskeleton	0.0058
Metabolism of xenobiotics by cytochrome P450	0.0071
Autophagy	0.0101
Gastric acid secretion	0.0103
Pancreatic cancer	0.0103
Dopaminergic synapse	0.0103
RNA transport	0.0112

**Alcohol DA**	**adjusted p value**
Upregulated	
Alcoholism	0.0117
Fatty acid degradation	0.0118
Axon guidance	0.0121
Long-term potentiation	0.0121
Salmonella infection	0.0133
Oxytocin signaling pathway	0.0134
Bacterial invasion of epithelial cells	0.0135
Chemokine signaling pathway	0.0136
Fatty acid biosynthesis	0.0137
Proteasome	0.0138
GABAergic synapse	0.0142
Ras signaling pathway	0.0157
Phospholipase D signaling pathway	0.0159
Gap junction	0.0165
Colorectal cancer	0.0165
VEGF signaling pathway	0.0171
Proximal tubule bicarbonate reclamation	0.0171
Epstein-Barr virus infection	0.0172
Pentose phosphate pathway	0.0174
AMPK signaling pathway	0.0175
Phagosome	0.0177
Platelet activation	0.0179
Drug metabolism	0.0190
Endocrine and other factor-regulated calcium reabsorption	0.0194
FoxO signaling pathway	0.0199
Hippo signaling pathway	0.0208
Ubiquitin mediated proteolysis	0.0216
Drug metabolism	0.0227
Complement and coagulation cascades	0.0233
Glycerophospholipid metabolism	0.0235
Fluid shear stress and atherosclerosis	0.0236
Proteoglycans in cancer	0.0254
Morphine addiction	0.0257
NOD-like receptor signaling pathway	0.0258
Ferroptosis	0.0266
Rap1 signaling pathway	0.0277
Cytokine-cytokine receptor interaction	0.0304
PD-L1 expression and PD-1 checkpoint pathway in cancer	0.0337
Focal adhesion	0.0339
Non-small cell lung cancer	0.0342
Vasopressin-regulated water reabsorption	0.0343
Sphingolipid signaling pathway	0.0344
Fc gamma R-mediated phagocytosis	0.0360
Signaling pathways regulating pluripotency of stem cells	0.0425
Wnt signaling pathway	0.0447
Staphylococcus aureus infection	0.0489

Supplementary Figure S3. Functional enrichment analysis of upregulated DEGs. DEG lists following perinatal alcohol on DA neurons. GO biological processes and KEGG pathway analysis results are listed in the accompanied tables with BH-corrected p-values.

Alcohol (non-DA) Upregulated

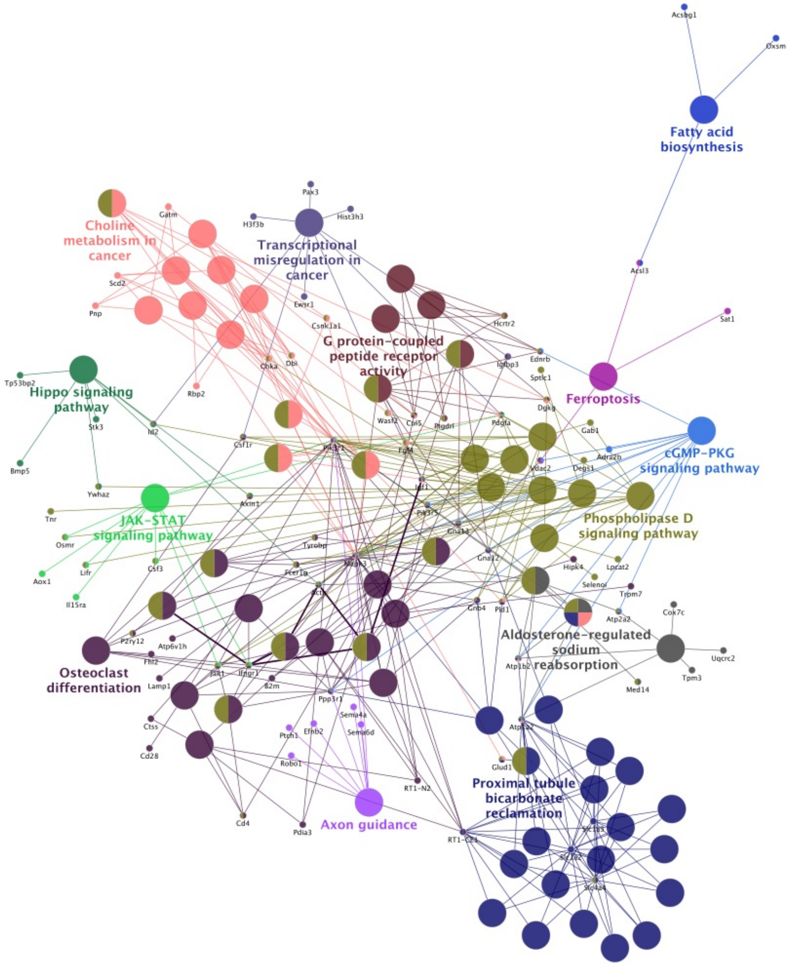

Alcohol NDA	adjusted p value
Upregulated	
Aldosterone-regulated sodium reabsorption	0.0114
Phospholipase D signaling pathway	0.0117
Proximal tubule bicarbonate reclamation	0.0148
cGMP-PKG signaling pathway	0.0154
Choline metabolism in cancer	0.0159
Osteoclast differentiation	0.0162
PD-L1 expression and PD-1 checkpoint pathway in cancer	0.0165
Sphingolipid signaling pathway	0.0179
Regulation of actin cytoskeleton	0.0179
G protein-coupled peptide receptor activity	0.0183
Thyroid hormone signaling pathway	0.0184
G protein-coupled receptor activity	0.0185
G protein-coupled receptor signaling pathway	0.0188
Transcriptional misregulation in cancer	0.0188
Cardiac muscle contraction	0.0189
Ras signaling pathway	0.0191
Cellular lipid metabolic process	0.0194
Response to drug	0.0195
Organic anion transmembrane transporter activity	0.0195
Organic substance biosynthetic process	0.0196
JAK-STAT signaling pathway	0.0197
Cellular senescence	0.0198
Platelet activation	0.0198
Lipid biosynthetic process	0.0202
PI3K-Akt signaling pathway	0.0205
Natural killer cell mediated cytotoxicity	0.0205
Regulation of localization	0.0206
Human cytomegalovirus infection	0.0209
Fatty acid biosynthesis	0.0211
Lipid metabolic process	0.0211
Melanoma	0.0216
Signaling pathways regulating pluripotency of stem cells	0.0217
Antigen processing and presentation	0.0219
Monovalent inorganic cation transport	0.0229
Human immunodeficiency virus 1 infection	0.0230
Active ion transmembrane transporter activity	0.0234
Signal transduction	0.0241
Cellular biosynthetic process	0.0242
Kaposi sarcoma-associated herpesvirus infection	0.0243
Regulation of transport	0.0247
Anion transmembrane transporter activity	0.0247
Inorganic cation transmembrane transport	0.0252
Rap1 signaling pathway	0.0252
Anion transmembrane transport	0.0261
Axon guidance	0.0272
Organophosphate metabolic process	0.0275
Organic anion transport	0.0275
Inorganic ion transmembrane transport	0.0276

Alcohol NDA	adjusted p value
Upregulated	
Glutamatergic synapse	0.0278
Tuberculosis	0.0279
Monovalent inorganic cation transmembrane transporter activity	0.0297
Ion transmembrane transport	0.0301
Active transmembrane transporter activity	0.0317
Organonitrogen compound metabolic process	0.0318
Hippo signaling pathway	0.0323
Regulation of signal transduction	0.0326
Long-term depression	0.0367
Th1 and Th2 cell differentiation	0.0373
Cation transmembrane transport	0.0393
Transmembrane transport	0.0423
Phosphorylation	0.0441
Glycerophospholipid metabolism	0.0441
Ion transport	0.0468
Cation transport	0.0483
Phosphate-containing compound metabolic process	0.0487
Ferroptosis	0.0487
Anion transport	0.0487

Supplementary Figure S4. Functional enrichment analysis of upregulated DEGs. DEG lists following perinatal alcohol on non-DA neurons exposure. GO biological processes and KEGG pathway analysis results are listed in the accompanied tables with BH-corrected p-values.

Nicotine-Alcohol (DA) Downregulated

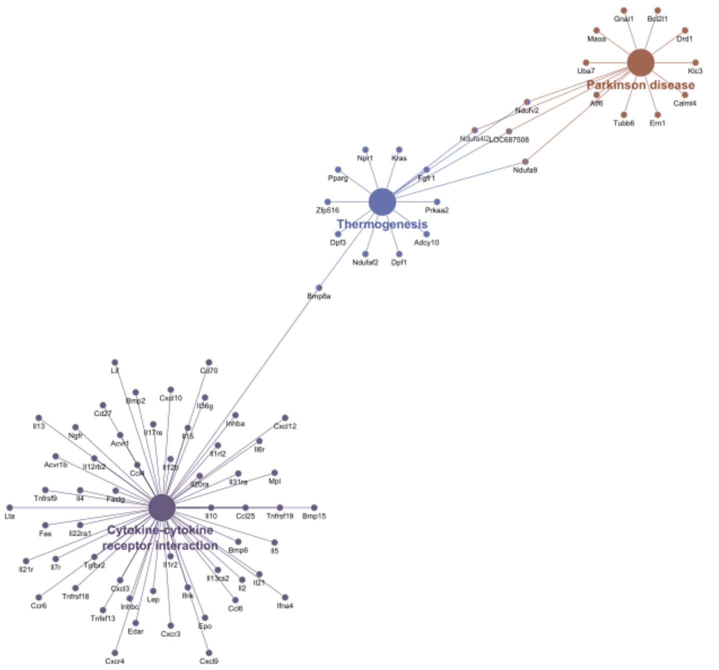

**Nicotine-Alcohol DA *Downregulated***	**adjusted p value**
Parkinson disease	0.0092
Thermogenesis	0.0410
Cytokine-cytokine receptor interaction	0.0413

Supplementary Figure S5. Functional enrichment analysis of downregulated DEGs. DEG lists following perinatal nicotine-alcohol on DA neurons exposure. GO biological processes and KEGG pathway analysis results are listed in the accompanied tables with BH-corrected p-values.

Nicotine-Alcohol (non-DA) Downregulated

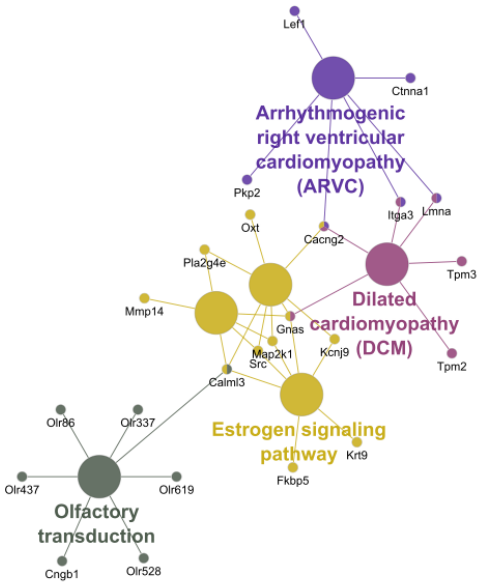

**Nicotine-Alcohol NDA**	**adjusted p value**
Downregulated	
Olfactory transduction	0.0062
Arrhythmogenic right ventricular cardiomyopathy (ARVC)	0.0180
Dilated cardiomyopathy (DCM)	0.0224
Estrogen signaling pathway	0.0255
GnRH signaling pathway	0.0266
Oxytocin signaling pathway	0.0289

Supplementary Figure S6. Functional enrichment analysis of downregulated DEGs. DEG lists following perinatal nicotine-alcohol on non-DA neurons exposure. GO biological processes and KEGG pathway analysis results are listed in the accompanied tables with BH-corrected p-values.

Alcohol (DA) Downregulated

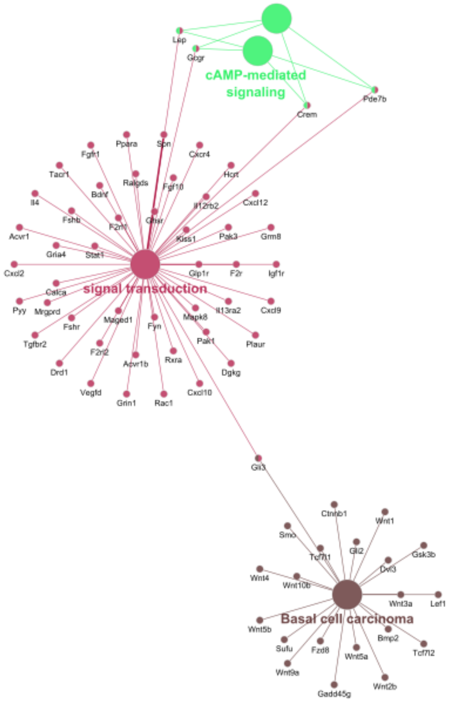

**Alcohol DA**	**adjusted p value**
Downregulated	
Basal cell carcinoma	0.0100
signal transduction	0.0142
cyclic-nucleotide-mediated signaling	0.0411
cAMP-mediated signaling	0.0411

Supplementary Figure S7. Functional enrichment analysis of downregulated DEGs. DEG lists following perinatal alcohol on DA neurons exposure. GO biological processes and KEGG pathway analysis results are listed in the accompanied tables with BH-corrected p-values.

Alcohol (non-DA) Downregulated

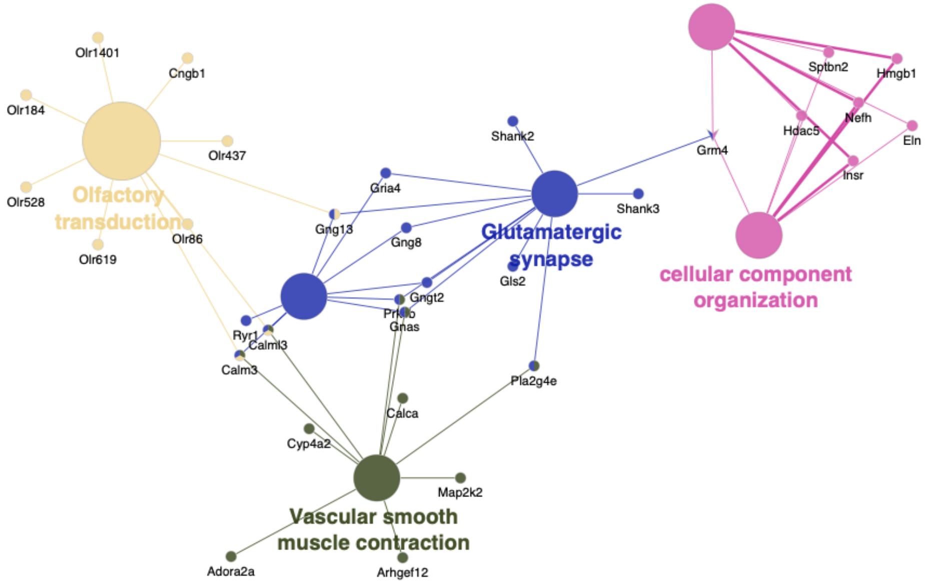

**Alcohol NDA**	**adjusted p value**
Downregulated	
Olfactory transduction	1.75E-06
Glutamatergic synapse	0.0123
Circadian entrainment	0.0307
cellular component organization or biogenesis	0.0397
cellular component organization	0.0397
Vascular smooth muscle contraction	0.0490

Supplementary Figure S8. Functional enrichment analysis of downregulated DEGs. DEG lists following perinatal alcohol on non-DA neurons exposure. GO biological processes and KEGG pathway analysis results are listed in the accompanied tables with BH-corrected p-values.

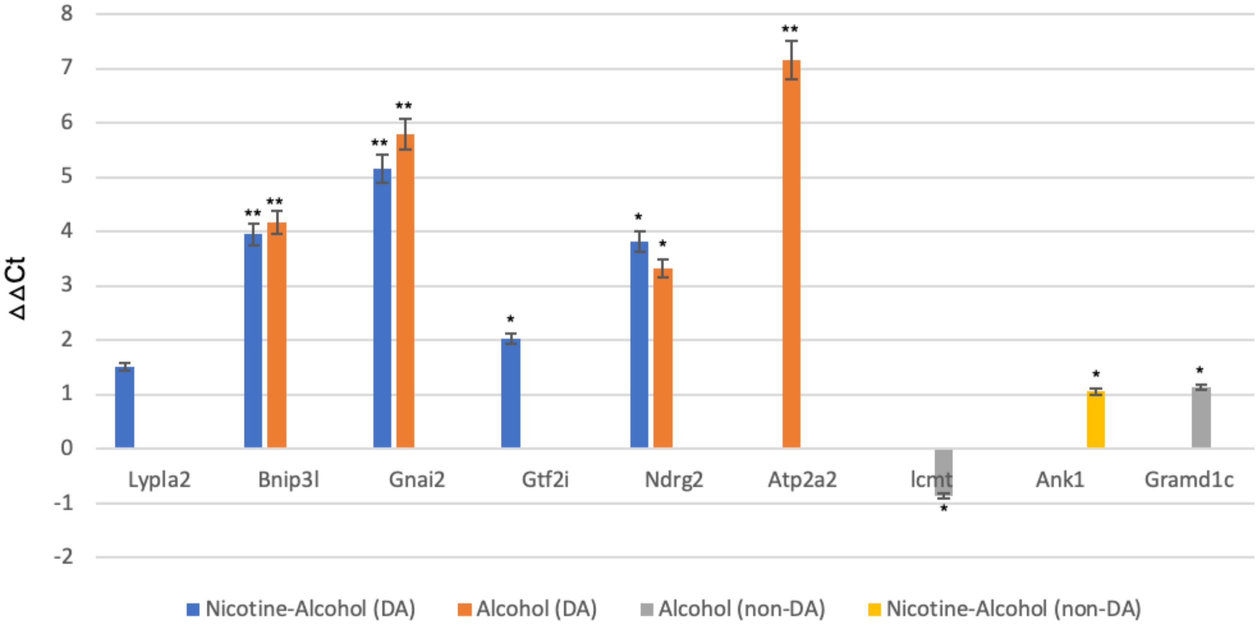

Supplementary Figure S9. Validation results by RT-qPCR. DEGs following perinatal nicotine-alcohol on DA neurons, alcohol on DA neurons, nicotine-alcohol on non-DA neurons, and alcohol on non-DA neurons exposure. Results are shown as ΔΔCt values relative to saline-counterpart and reference primer GAPDH. Significance was evaluated using Student's t-test (n=3). *denotes $p<0.05$, **denotes $p<0.01$.
